# Role of Lung Function, Chronic Obstructive Pulmonary Disease on Hearing Impairment: Evidence for Causal Effects and Clinical Implications

**DOI:** 10.3390/audiolres15040088

**Published:** 2025-07-16

**Authors:** Lanlai Yuan, Feipeng Cui, Ge Yin, Mengwen Shi, Nadida Aximu, Yaohua Tian, Yu Sun

**Affiliations:** 1 Department of Otolaryngology Head and Neck Surgery, Sichuan Provincial People’s Hospital, University of Electronic Science and Technology of China, Chengdu 610072, China; yuanlanlai@med.uestc.edu.cn; 2 Department of Otorhinolaryngology, Union Hospital, Tongji Medical College, Huazhong University of Science and Technology, Wuhan 430022, China; d202381984@hust.edu.cn (G.Y.); d202482323@hust.edu.cn (M.S.); 3 Ministry of Education Key Laboratory of Environment and Health, and State Key Laboratory of Environmental Health (Incubating), School of Public Health, Tongji Medical College, Huazhong University of Science and Technology, Wuhan 430030, China; d202181624@hust.edu.cn (F.C.); yaohua_tian@hust.edu.cn (Y.T.); 4 Department of Maternal and Child Health, School of Public Health, Tongji Medical College, Huazhong University of Science and Technology, Wuhan 430030, China; 5 School of Public Health, Xinjiang Medical University, Urumqi 830000, China; nadida123@163.com; 6 Institute of Otorhinolaryngology, Union Hospital, Tongji Medical College, Huazhong University of Science and Technology, Wuhan 430022, China; 7 Hubei Province Clinical Research Center for Deafness and Vertigo, Wuhan 430022, China

**Keywords:** hearing impairment, lung function, chronic obstructive pulmonary disease, COPD, Mendelian randomization

## Abstract

**Objectives**: Observational studies have shown that chronic obstructive pulmonary disease (COPD) is associated with an increased risk of hearing impairment. However, causality remains unclear, including with respect to lung function. This study aimed to investigate the associations of lung function and COPD with hearing impairment in the UK Biobank and confirm potential causalities using Mendelian randomization (MR). **Methods:** Cross-sectional analyses were performed using logistic regression models in a subsample of the UK Biobank. Two-sample MR analyses were performed on summary statistics for forced expiratory volume in one second (FEV1), forced vital capacity (FVC), COPD, and sensorineural hearing loss. **Results**: FEV1 and FVC were negatively associated with hearing impairment, with odds ratios (95% confidence intervals) of 0.80 (0.77, 0.84) and 0.80 (0.76, 0.83), respectively. COPD was positively associated with hearing impairment, with an odds ratio (95% confidence interval) of 1.10 (1.02, 1.18). In the MR analyses, a negative association was found between FVC and sensorineural hearing loss, with an odds ratio (95% confidence interval) of 0.91 (0.83, 0.99). For FVE1 and COPD, no significant associations were found. **Conclusions**: The results of this study showed that FVC was causally associated with hearing impairment, suggesting a potential protective effect of FVC on hearing impairment.

## 1. Introduction

Hearing impairment is a condition that is increasingly common with age [1]. According to the World Health Organization in 2021, more than 1.5 billion people worldwide suffered from hearing impairment [2]. By 2050, this number could rise to 2.5 billion [2]. Hearing impairment can adversely affect an individual in many ways, such as reduced quality of life, limited employment opportunities, and increased risk of cognitive decline, dementia, loneliness, isolation, and depression [3]. Identified risk factors associated with hearing impairment include age, genetic factors, noise exposure, modifiable lifestyle (e.g., smoking, and diet), and chronic diseases (e.g., obesity, diabetes, and cardiovascular disease) [2]. Importantly, many of the risk factors for hearing impairment are preventable, and altering modifiable risk factors can change the course of an individual’s hearing trajectory and affect the extent of hearing impairment in later life [2]. Therefore, identifying modifiable risk factors to prevent hearing impairment is of great importance.

Chronic obstructive pulmonary disease (COPD) has been reported to be associated with hearing impairment or sensorineural hearing loss in several previous observational studies [4,5,6]. The pathology of COPD is characterized by airway obstruction that is incompletely reversible, usually progressive, and associated with inflammation [7]. Hypoxemia and chronic systemic inflammation have been hypothesized as underlying mechanisms in the association of COPD with hearing impairment [4,5,6]. However, causality remains uncertain, as the association between COPD and hearing impairment may be influenced by residual confounding or reverse causal association. In addition, there is a lack of studies investigating the association between lung function and hearing impairment. Given that the decline in lung function in adults may be preventable in some cases [8], establishing a causal association between lung function and hearing impairment may be important in preventing hearing impairment.

Compared to conventional observational studies, Mendelian randomization (MR) studies may provide more reliable evidence of causal relationships between lung function and COPD with hearing impairment. MR uses genetic variation as the instrumental variable (IV) for exposure to provide evidence of causal relationships between modifiable risk factors and diseases [9]. The alleles of genetic variation are randomly assigned to sperm or egg cells during human gametogenesis, independently of potentially confounding environmental exposures [10,11]. Additionally, the genetic variation is fixed in nature, which ensures a lifetime of exposure and mitigates concerns about reverse causality [10]. Therefore, MR can reduce the potential biases caused by confounding and reverse causality [11]. This study aimed to investigate the associations of lung function and COPD with hearing impairment using large cross-sectional data from the UK Biobank cohort. In addition, two-sample MR was used to confirm potential causal associations.

## 2. Materials and Methods

### 2.1. Study Design and Population

The UK Biobank is a national prospective cohort that enrolled over half a million participants aged 40 to 69 years from 22 assessment centers across the UK between 2006 and 2010. This cohort was designed to study lifestyle, genetic, and environmental factors contributing to various diseases in middle and old age. A wide range of information was collected from participants, including questionnaires, physical measurements, biological samples, imaging, and follow-up on a variety of health-related outcomes. More details about the UK Biobank cohort are given elsewhere [12]. Written informed consent was obtained from all participants, and ethical approval for the UK Biobank was granted by the North West Multicenter Research Ethics Committee. The study followed the tenets of the Declaration of Helsinki.

**Figure 1:**
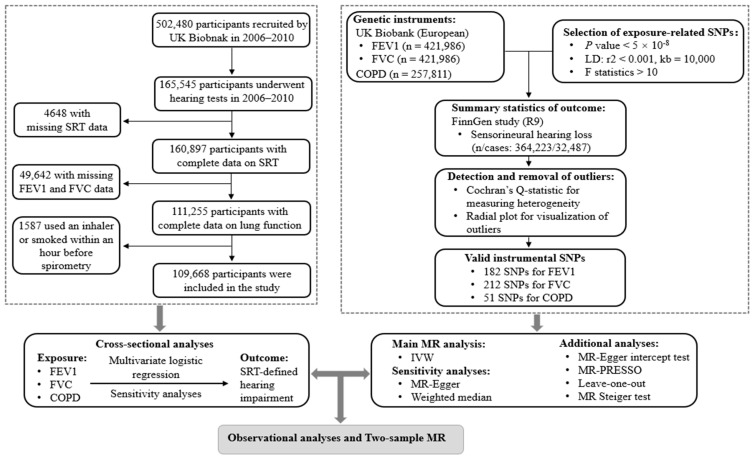
Flow chart and study design. SRT, speech reception threshold; FEV1, forced expiratory volume in one second; FVC, forced vital capacity; COPD, chronic obstructive pulmonary disease; SNPs, single-nucleotide polymorphisms; LD, linkage disequilibrium; MR, Mendelian randomization; IVW, inverse-variance weighted; MR-PRESSO, Mendelian randomization pleiotropy residual sum and outlier.

Figure 1 shows a flowchart of the study participants. There were 165,545 participants in the UK Biobank cohort who underwent a hearing test at baseline. After excluding missing data for hearing tests (*n* = 4648) and lung function (n = 49,642), followed by further exclusion of participants who smoked or used an inhaler within one hour before the spirometry (*n* = 1587), a total of 109,668 participants of European ancestry were included in the cross-sectional analyses.

### 2.2. Spirometry and Definition of COPD

At the 5th station of the assessment center visit of the UK Biobank, pre-bronchodilator spirometry (Vitalograph Pneumotrac 6800) was performed. Two to three breaths were recorded for each participant for approximately six minutes, with each blow lasting at least six seconds. The reproducibility of the first two blows was compared by computer. If the difference in forced expiratory volume in one second (FEV1) and forced vital capacity (FVC) was less than 5%, it was considered acceptable, indicating that a third blow was not required. The highest FVC and FEV1 values were used in this study.

According to the definition of COPD used in a previous study [13], participants were defined as having COPD if the FEV1/FVC ratio was less than the lower limit of normal (LLN) calculated based on age, sex, race (Caucasian), and height using the Global Lung Function Initiative 2012 equations [14]. In a sensitivity analysis, COPD was defined as an FEV1/FVC ratio below 0.7.

### 2.3. Definition of Hearing Impairment

A digit triplet test (DTT) was used for hearing tests in the UK Biobank. Fifteen sets of monosyllabic English digits were presented to the participants over background noise. An increase in the level of subsequent background noise would follow if participants correctly identified the triplet and a decrease if they did not. The signal-to-noise ratio with half of the speech delivered correctly understood was used to define the speech recognition threshold (SRT). The range of the SRT was from −12 to +8 dB, with the lower values representing better hearing ability. Using cut-off points established in a previous study [15], participants in this study were divided into two groups based on the better ear: normal hearing (SRT < −5.5 dB) and hearing impairment (SRT ≥ −5.5 dB).

### 2.4. Covariates

A series of covariates (potential confounders) were used in this study according to previous studies. Covariates are described in more detail in Supplemental Text S1.

### 2.5. Data Sources and Genetic Instruments for MR Analyses

The two-sample MR using genome-wide association study (GWAS) summary statistics was performed to estimate the causal effects of lung function and COPD (exposure) on sensorineural hearing loss (outcome). Figure 1 outlines the study design for the two-sample MR. Valid genetic IVs in MR studies satisfy three key assumptions: (1) relevance: the IVs are strongly associated with the exposure; (2) independence: the IVs are not associated with confounding factors in the link between exposure and outcome; and (3) exclusion restriction: the IVs are exclusively associated with the outcome because of their effect on the exposure [9].

Details of the GWAS summary statistics are provided in Table S1, with no overlap in the sample population for exposure and outcome. All GWAS summary data used in this study had received participants’ informed consent and ethical approval in the original study. Details of the data sources and selection of genetic instruments are provided in Supplemental Text S2.

Outliers were detected using radial plots and excluded before the primary MR analyses (Figures S1–S3). The advantage of radial plots is that they improve the visual detection of influential data points and outliers [16]. As a result, 182 single-nucleotide polymorphisms (SNPs) associated with FVE1, 212 SNPs associated with FVC, and 51 SNPs associated with COPD were used for the MR analyses. More information on these SNPs is provided in Tables S2–S4.

### 2.6. Statistical Analyses

Details of statistical analyses are provided in Supplemental Text S3.

#### 2.6.1. Cross-Sectional Analyses

The associations of lung function and COPD with the risk of hearing impairment were analyzed using multivariate logistic regression models, with the results expressed as odds ratios (ORs) and corresponding 95% confidence intervals (CIs).

#### 2.6.2. Two-Sample MR

In the primary MR analyses, an inverse-variance weighted (IVW) method was used as the main method to assess the potential causal effects of lung function and COPD on hearing impairment (i.e., sensorineural hearing loss). If all IVs are valid, IVW yields the most precise estimate. MR-Egger regression and weighted median were used as sensitivity analyses.

All analyses were conducted using the statistical software R (version 4.2.3). The R packages TwoSampleMR, RadialMR, and MRPRESSO were used for the MR analyses. Two-sided *p* values below 0.05 were used as the statistical significance level.

## 3. Results

### 3.1. Associations of Lung Function and COPD with Hearing Impairment in UK Biobank

Table 1 and Table 2 summarize the basic characteristics of the study population. A total of 109,668 participants were included in the cross-sectional analyses, including 10,900 (9.9%) participants with SRT-defined hearing impairment and 98,768 (90.1%) participants with normal hearing. The median (interquartile range) age was 58.00 (12.00) years, and 58,994 (53.8%) participants were female. Participants with hearing impairment were older, more likely to be male, and of lower socioeconomic status than those with normal hearing.

Table 3 shows the results of the logistic regression analyses. After adjustment for potential confounders, lung function was associated with a reduced risk of hearing impairment, with ORs (95% CIs) for each interquartile range increase in FEV1 and FVC of 0.80 (0.77, 0.84) and 0.80 (0.76, 0.83), respectively. Results stratified by age and sex displayed similar associations (*p* for interactions > 0.05) (Table S5). Compared to participants without COPD, those with COPD (FEV1/FVC < LLN) had an increased risk of hearing impairment, with an OR (95% CI) of 1.10 (1.02, 1.18) (Table 3). This significant association was observed in participants aged 60 years or older, but not in those under 60 years (*p* for interaction > 0.05) (Table S5). Moreover, the association between COPD (FEV1/FVC < LLN) and increased risk of hearing impairment was observed in both men and women, but without statistical significance (*p* for interaction > 0.05) (Table S5).

In the sensitivity analysis, a similar result was observed for COPD, which was defined as an FEV/FVC ratio below 0.7 (Table S6). Excluding participants with a history of asthma did not significantly change the result (Table S7). In the restricted cubic spline models, the associations of FEV1 and FVC with hearing impairment were linear (*p* for nonlinear > 0.05) (Figures S4 and S5). When self-reported hearing difficulty was treated as a secondary outcome, the results for FEV1 and FVC remained significant, but no significant association was observed for COPD (Tables S8 and S9).

### 3.2. Results for MR Analyses

**Figure 2:**
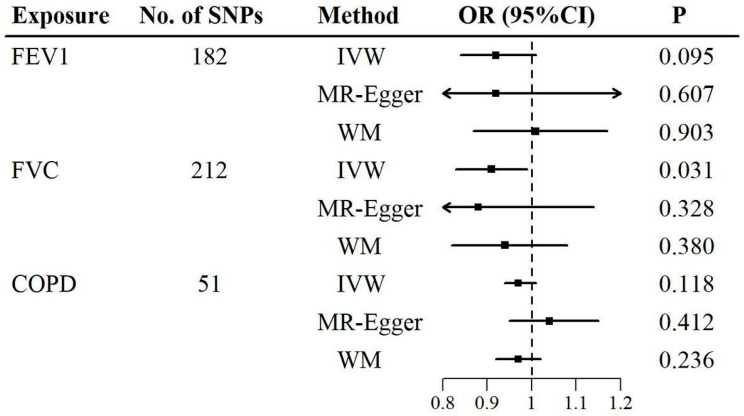
Two-sample Mendelian randomization analyses of the associations of lung function and COPD with sensorineural hearing loss after excluding radial IVW outliers. No., number; SNPs, single-nucleotide polymorphisms; OR, odds ratio; CI, confidence interval; IVW, inverse-variance weighted; WM, weighted median; FEV1, forced expiratory volume in one second; FVC, forced vital capacity; COPD, chronic obstructive pulmonary disease. For lung function, the results were reported as ORs for sensorineural hearing loss per standard deviation increase in FEV1 and FVC.

The causal associations of lung function and COPD with sensorineural hearing loss were determined using the two-sample MR (Figure 2, Tables S10 and S11). In the primary MR analyses (outliers were excluded), the IVW method showed a negative association between FVC and sensorineural hearing loss, with an OR (95% CI) of 0.91 (0.83, 0.99) per standard deviation increase in FVC (Figure 2 and Table S10). In the sensitivity analyses, the MR-Egger and weighted median methods were similar to the IVW method in direction and magnitude, with ORs (95% CIs) of 0.88 (0.68, 1.14) and 0.94 (0.82, 1.08) per standard deviation increase in FVC, respectively (Figure 2 and Table S10). However, no significant causal association was found between FEV1 and sensorineural hearing loss in either the IVW method or the sensitivity analyses (Figure 2 and Table S10). For COPD, there was also no evidence of a causal association (Figure 2 and Table S10). 

The Cochran’s Q statistic, scatter plots, and funnel plots suggested that no substantial heterogeneity was found in the primary MR analyses (Table S12 and Figures S6–S11). No evidence of horizontal pleiotropy was shown in the MR-Egger intercept or Mendelian randomization pleiotropy residual sum and outlier (MR-PRESSO) test (Tables S13 and S14). The leave-one-out plot showed that the estimate of the overall significance of FVC was not driven by any single SNP (Figure S12). The results of the MR Steiger test did not support the reverse causal association between FVC and sensorineural hearing loss (Table S15).

## 4. Discussion

In the cross-sectional analyses of 109,668 middle-aged and older adults in the UK Biobank cohort, better lung function (including FEV1 and FVC) was associated with a reduced risk of hearing impairment, and this association showed a linear dose–response relationship. COPD was positively associated with hearing impairment. In addition, the primary MR analysis (IVW) found a significant negative association between FVC and sensorineural hearing loss, which was robust to sensitivity analyses, heterogeneity, and pleiotropy tests, indicating a potential protective effect. For FEV1 and COPD, however, there were no significant associations with sensorineural hearing loss in the MR analyses.

At present, data on the association between lung function and hearing impairment are lacking. In this study, the observational and primary MR analyses yielded consistent results of a significant negative association between FVC and hearing impairment, while no statistical significance was shown for FEV1 in the MR analyses. These findings suggested that FEV1 and FVC might differ in their associations with hearing impairment. The most common measures used in spirometry are FEV1 and FVC, which reflect different assessments of lung function [17,18]. FEV1 is used to measure airway obstruction, while FVC measures total lung capacity [19]. Age, gender, race, height, and smoking history were the most important predictors of lung function, with height highly linked with FVC and smoking history strongly associated with FEV1 [18,19]. Therefore, one possible explanation for the discrepancy in the results could be a spurious association between FEV1 and hearing impairment due to residual confounding in observational studies that lacked complete information on the number, frequency, intensity, and cessation of smoking over the life course. In contrast, MR studies are based on the principle that genotypes in a population are generally independent of confounders, making the results less affected by confounding and providing more reliable evidence when MR assumptions are adequately met [9,10,11]. For FVC instead of FEV1, similar magnitudes and consistent directions were estimated by the IVW, MR-Egger, and weighted median methods. This suggested a potential protective effect of FVC on hearing impairment.

Although many observational studies have found that impaired lung function (including FEV1 and FVC) is associated with mortality and multiple adverse health outcomes, FVC appears to be the more relevant determinant [20,21,22,23,24]. Even without chronic lung disease, the reduced FVC, a sign of lung function restriction, was a strong predictor of mortality [25,26]. Only FVC was still strongly associated with mortality when FEV1 and FVC were further adjusted for each other in the model [25]. Another study revealed true lung restriction in people who had only a low FVC trajectory (restrictive pattern only), with a risk of multiple diseases by middle age [27]. Similarly, compared to the obstructive pattern (reduced FEV1/FVC), the restrictive pattern of impaired lung function (reduced percent predicted FVC) was a stronger predictor of incident chronic kidney disease and end-stage renal disease [28]. These were further supported by a recent MR study in which reduced FVC had an independent causal association with coronary artery disease [23]. Conversely, there was little evidence to support a causal link between FEV1 and the risk of cardiovascular disease [23]. In line with previous studies, this study found a causal association of FVC (rather than FEV1) with hearing impairment.

On the other hand, the underlying mechanism of the association between FVC and hearing impairment could possibly be explained by the independent and stronger association of FVC with multiple diseases [29], such as cardiovascular disease, diabetes, and metabolic syndrome, which have been linked to hearing impairment [2]. Furthermore, there is growing evidence that impaired lung function in adults may be due in part to poor growth and development and associated with various risk factors throughout the life course, such as maternal smoking, prematurity, low birth weight, physical inactivity, early smoking, or exposure to air pollution [24,26]. As suggested by the restrictive-only pattern, poor lung development seemed to be linked to the underdevelopment of other organ systems and subsequent multiple morbidities [27]. This implied that a causal association between FVC and hearing impairment might involve shared pathophysiological pathways. Mechanistically, the causal association between FVC and hearing impairment could be explained by hypoxia and chronic systemic inflammation. Specifically, impaired lung function may lead to hypoxia [30], triggering chronic systemic inflammation [31], thereby directly or indirectly resulting in hearing impairment [32,33,34]. However, the specific mechanisms behind this causal association remained unknown and required additional research.

An association between COPD and hearing impairment has been reported in several previous observational studies. A meta-analysis showed that patients with COPD had significantly prolonged auditory brainstem response waves and significantly elevated pure tone audiometry than the controls [4]. In another cross-sectional study based on the National Health and Nutrition Examination Survey, an independent association between COPD and sensorineural hearing loss was found after adjustment for many confounders [5]. Moreover, a large cohort study reported that COPD was associated with low- and mid-frequency hearing decline [6]. By contrast, the MR analyses in this study did not show that genetically predicted COPD was associated with an increased risk of hearing impairment, suggesting that the significant associations reported in the observational studies might be due to residual confounding or reverse associations. However, a causal effect of COPD on hearing impairment could not be completely ruled out, due to the insufficient statistical power resulting from the use of a limited number of genetic IVs that might only explain a small proportion of the heterogeneous phenotypes of COPD. This may be clarified by obtaining more genetic IVs for COPD in the future.

A strength of this study was the large sample size. Another strength was that the MR analyses provided causal inference, with sensitivity analyses, heterogeneity, and pleiotropy tests showing no violation of assumptions. In addition, the reverse causality risk was reduced by the MR Steiger test.

However, this study also had several limitations. Firstly, lung function generally varies with time [28]. More insight may be provided by further investigation of the longitudinal association between lung function trajectories and hearing impairment. Secondly, the definition of COPD should be based on post-bronchodilator spirometry. However, only pre-bronchodilator spirometry was carried out in the UK Biobank, which could result in the misclassification of exposure. Using pre-bronchodilator spirometry to define COPD could not rule out reversible airway obstruction, the most common cause of which is asthma. Nevertheless, excluding participants with a history of asthma did not significantly change the main results. Thirdly, although MR analyses of lung function were performed with a sufficient set of genetic IVs, the number of genetic IVs was relatively small for COPD, which might reduce the statistical power. Fourthly, the definition of outcome in the observational study was not consistent with that in the MR analyses. The former was based on the SRT obtained from the DTT in the UK Biobank, and the latter was based on ICD-9 and ICD-10 codes (sensorineural hearing loss: ICD-9 [3891] and ICD-10 [H90.3, H90.4, H90.5]) in the FinnGen study. Notably, a previous study has suggested that the DTT is a signal-to-noise ratio test that can overcome conduction loss by turning up the volume of the stimuli, as allowed for participants in the UK Biobank [35]; that is, the DTT is a sensorineural hearing loss test that detects hearing impairment with a cochlear origin only [35]. However, no further audiological assessments were performed in the UK Biobank, such as the collection of any data on conductive pathologies, otoscopic findings, or measures of middle ear function (https://www.ukbiobank.ac.uk/, accessed on 30 July 2023). Consequently, we were not able to completely exclude those with conductive hearing loss. The results of our MR analyses should be interpreted with caution, as the potential causal effects may be limited to sensorineural hearing loss. Fifthly, given the complexity of hearing impairment pathogenesis, the specific role of FVC could not be determined. To elucidate the underlying mechanisms, further experimental studies are needed to confirm the biological rationale and infer causality. Sixthly, while the MR Steiger test supported the absence of reverse causality between lung function and sensorineural hearing loss, a plausible biological pathway could theoretically exist whereby early-life hearing impairment might affect lung development via its impact on physical activity. This could potentially bias the association between genetically predicted lung function and hearing impairment. However, there is currently a lack of individual-level data and GWAS summary statistics on early-life hearing impairment and physical activity during childhood or adolescence. Longitudinal mediation analyses and two-stage MR analyses could be used to validate this biological pathway when appropriate data are available. Finally, the study population was predominantly of European ancestry, which limited the generalization of our results to other populations.

## 5. Conclusions

In conclusion, a linear negative association was found between FVC and hearing impairment in middle-aged and older adults in the UK Biobank. In addition, MR analyses confirmed a causal effect of FVC on hearing impairment, but not FEV1 and COPD. Given that spirometry is a non-invasive, simple, and reproducible method, early, especially in key age windows such as childhood and adolescence [24], and regular testing of lung function can help identify individuals at risk of hearing impairment, allowing for timely interventions and optimal management. From a clinical perspective, it is recommended that early hearing screening be incorporated into the clinical management of patients with decreased lung function or COPD. Once hearing impairment is identified, close collaboration between respiratory physicians and otolaryngologists or audiologists is essential, along with prompt referrals for support with hearing technology, such as hearing aids.

## Figures and Tables

**Table 1:** Demographic characteristics of study participants.

		Hearing Impairment		p *
	Total	No	Yes	
No. of participants	109,668	98,768	10,900	
Age (years), median (IQR)	58.00 (12.00)	58.00 (13.00)	63.00 (8.00)	<0.001
FEV1 (liters), mean (SD)	2.85 (0.75)	2.88 (0.75)	2.65 (0.71)	<0.001
FVC (liters), mean (SD)	3.76 (0.94)	3.79 (0.94)	3.54 (0.90)	<0.001
Sex (%)				0.022
Male	50,674 (46.2)	45,524 (46.1)	5150 (47.2)	
Female	58,994 (53.8)	53,244 (53.9)	5750 (52.8)	
TDI (%)				<0.001
1st	27,346 (24.9)	24,883 (25.2)	2463 (22.6)	
2nd	27,398 (25.0)	24,682 (25.0)	2716 (24.9)	
3rd	27,378 (25.0)	24,749 (25.1)	2629 (24.1)	
4th	27,375 (25.0)	24,305 (24.6)	3070 (28.2)	
Missing	171 (0.2)	149 (0.2)	22 (0.2)	
Qualifications (%)				<0.001
College or university degree	37,694 (34.4)	34,767 (35.2)	2927 (26.9)	
A levels or AS levels	13,058 (11.9)	12,053 (12.2)	1005 (9.2)	
or equivalent				
O levels, GCSEs, or CSEs	30,350 (27.7)	27,679 (28.0)	2671 (24.5)	
or equivalent				
HND, HNC, NVQ, or other	12,673 (11.6)	11,168 (11.3)	1505 (13.8)	
professional qualification				
None of the above	15,141 (13.8)	12,476 (12.6)	2665 (24.4)	
Missing	752 (0.7)	625 (0.6)	127 (1.2)	
Employment (%)				<0.001
Employed	62,668 (57.1)	58,388 (59.1)	4280 (39.3)	
Retired	37,935 (34.6)	32,236 (32.6)	5699 (52.3)	
Other	8743 (8.0)	7855 (8.0)	888 (8.1)	
Missing	322 (0.3)	289 (0.3)	33 (0.3)	

* The *t*-tests or Wilcoxon rank sum tests were used for continuous variables and the Chi-square tests for categorical variables. IQR, interquartile range; SD, standard deviation; FEV1, forced expiratory volume in one second; FVC, forced vital capacity; TDI, Townsend deprivation index; GCSEs, general certificate of secondary education; CSEs, certificate of secondary education; HND, higher national diploma; HNC, higher national certificate; NVQ, national vocational qualification.

**Table 2:** Clinical characteristics of study participants.

	Total	Hearing Impairment		p *
	(*n* = 109,668)	No (*n* = 98,768)	Yes (*n* = 10,900)	
Drink frequency (%)				<0.001
Never	6724 (6.1)	5828 (5.9)	896 (8.2)	
Special occasions only	11,420 (10.4)	9959 (10.1)	1461 (13.4)	
One to three times a month	12,277 (11.2)	11,077 (11.2)	1200 (11.0)	
Once or twice a week	28,443 (25.9)	25,680 (26.0)	2763 (25.3)	
Three or four times a week	26,572 (24.2)	24,259 (24.6)	2313 (21.2)	
Daily or almost daily	24,177 (22.0)	21,915 (22.2)	2262 (20.8)	
Missing	55 (0.1)	50 (0.1)	5 (0.0)	
Smoking (%)				<0.001
Never	58,985 (53.8)	53,620 (54.3)	5365 (49.2)	
Previous	40,479 (36.9)	36,051 (36.5)	4428 (40.6)	
Current	9890 (9.0)	8838 (8.9)	1052 (9.7)	
Missing	314 (0.3)	259 (0.3)	55 (0.5)	
Body mass index, kg/m^2^ (%)				<0.001
<25	36,671 (33.4)	33,285 (33.7)	3386 (31.1)	
≥25 and <30	46,902 (42.8)	42,283 (42.8)	4619 (42.4)	
≥30	26,008 (23.7)	23,129 (23.4)	2879 (26.4)	
Missing	87 (0.1)	71 (0.1)	16 (0.1)	
Diabetes (%)				<0.001
No	104,431 (95.2)	94,317 (95.5)	10,114 (92.8)	
Yes	5046 (4.6)	4285 (4.3)	761 (7.0)	
Missing	191 (0.2)	166 (0.2)	25 (0.2)	
Cardiovascular diseases (%)				<0.001
None	78,963 (72.0)	71,921 (72.8)	7042 (64.6)	
Hypertension	23,176 (21.1)	20,413 (20.7)	2763 (25.3)	
Heart attack, angina,	2479 (2.3)	2092 (2.1)	387 (3.6)	
or stroke				
Hypertension, and heart	2469 (2.3)	2069 (2.1)	400 (3.7)	
attack, angina, or stroke				
Missing	2581 (2.4)	2273 (2.3)	308 (2.8)	
Work noise exposure (%)				<0.001
No	84,034 (76.6)	76,390 (77.3)	7644 (70.1)	
Yes	24,779 (22.6)	21,618 (21.9)	3161 (29.0)	
Missing	855 (0.8)	760 (0.8)	95 (0.9)	
Music noise exposure (%)				<0.001
No	94,773 (86.4)	85,158 (86.2)	9615 (88.2)	
Yes	13,475 (12.3)	12,350 (12.5)	1125 (10.3)	
Missing	1420 (1.3)	1260 (1.3)	160 (1.5)	
COPD				<0.001
No	101,332 (92.4)	91,383 (92.5)	9949 (91.3)	
Yes	8336 (7.6)	7385 (7.5)	951 (8.7)	

* The Chi-square tests for categorical variables. COPD, chronic obstructive pulmonary disease.

**Table 3:** Associations of lung function and COPD with the odds of hearing impairment (*n* = 109,668).

Exposure	Unadjusted OR (95% CI)	*p*	Adjusted * OR (95% CI)	*p*
FEV1, per IQR	0.64 (0.62, 0.65)	<0.001	0.80 (0.77, 0.84)	<0.001
FVC, per IQR	0.67 (0.65, 0.69)	<0.001	0.80 (0.76, 0.83)	<0.001
COPD (FEV1/FVC < LLN) ^†^	1.18 (1.10, 1.27)	<0.001	1.10 (1.02, 1.18)	0.012

The FEV1 of each IQR is equivalent to 1.04 L. The FVC of each IQR is equivalent to 1.33 L. * Adjusted for age, sex, Townsend deprivation index, qualifications, employment, smoking, drink frequency, body mass index, diabetes, cardiovascular diseases, and music and occupational noise exposure. ^†^ For COPD (FEV1/FVC < LLN), cases were 8336 (7.6%), controls were 101,332 (92.4%). OR, odds ratio; CI, confidence interval; FEV1, forced expiratory volume in one second; FVC, forced vital capacity; IQR, interquartile range; COPD, chronic obstructive pulmonary disease; LLN, lower limit of normal.

## Data Availability

All data supporting the reported results are publicly available. Data supporting the results of cross-sectional analyses are available by application from the UK Biobank website (www.ukbiobank.ac.uk, accessed on 30 July 2023). Summary data for FEV1 and FVC were obtained from the UK Biobank GWAS analyses available in the IEU GWAS database (https://gwas.mrcieu.ac.uk/, accessed on 20 October 2023). Summary data for COPD were contributed by Sakornsakolpat et al. Summary data for sensorineural hearing loss were downloaded from the FinnGen study (R9), available from the official website (https://www.finngen.fi/en, accessed on 19 October 2023).

## Supplementary Materials

The following supporting information can be downloaded at: https://www.mdpi.com/article/10.3390/audiolres15040088/s1; Supplemental Text S1–S3; Figure S1: A radial plot of the association between FEV1 and sensorineural hearing loss; Figure S2: A radial plot of the association between FVC and sensorineural hearing loss; Figure S3: A radial plot of the association between COPD and sensorineural hearing loss; Figure S4: Dose-response curve of FEV1 with odds of hearing impairment using restricted cubic spline model; Figure S5: Dose-response curve of FVC with odds of hearing impairment using restricted cubic spline model; Figure S6: Scatter plot of SNP-effect on FEV1 (x-axis) and SNP-effect on sensorineural hearing loss (y-axis); Figure S7: Scatter plot of SNP-effect on FVC (x-axis) and SNP-effect on sensorineural hearing loss (y-axis); Figure S8: Scatter plot of SNP-effect on COPD (x-axis) and SNP-effect on sensorineural hearing loss (y-axis); Figure S9: Funnel plot of the heterogeneity of the causal effect of FEV1 on sensorineural hearing loss; Figure S10: Funnel plot of the heterogeneity of the causal effect of FVC on sensorineural hearing loss; Figure S11: Funnel plot of the heterogeneity of the causal effect of COPD on sensorineural hearing loss; Figure S12: Leave-one-out analysis of the causal effect of FVC on sensorineural hearing loss after excluding radial IVW outliers; Table S1: Information of the genome wide association studies included in this study; Table S2: Genetic instruments for FEV1; Table S3: Genetic instruments for FVC; Table S4: Genetic instruments for COPD; Table S5: Associations of lung function and COPD with the odds of hearing impairment stratified by age and sex; Table S6: Association of COPD (FEV1/FVC < 0.7) with the odds of hearing impairment (*n* = 109,668); Table S7: Association of COPD with the odds of hearing impairment after excluding participants with a history of asthma (*n* = 96,160); Table S8: Associations of lung function and COPD with the odds of self-reported hearing difficulty (*n* = 333,082); Table S9: Associations of lung function and COPD with the odds of self-reported hearing difficulty after excluding participants with history of noise exposure (*n* = 299,670); Table S10: Two-sample Mendelian randomization analyses of the associations of lung function and COPD with sensorineural hearing loss after excluding radial IVW outliers; Table S11: Two-sample Mendelian randomization analyses of the associations of lung function and COPD with sensorineural hearing loss before excluding radial IVW outliers; Table S12: Heterogeneity statistics before and after excluding radial IVW outliers; Table S13: Results of the Egger-intercept test using the two-sample Mendelian randomization analysis after excluding radial IVW outliers; Table S14: Results of the MR-PRESSO test using the two-sample Mendelian randomization analysis after excluding radial IVW outliers; Table S15: Mendelian randomization Steiger directionality test for FVC. Refs. [9,11,16,36,37,38,39,40,41,42,43,44,45,46] are cited in the Supplementary Materials.

## Abbreviations

COPD: chronic obstructive pulmonary disease; CI: confidence interval; CSEs: certificate of secondary education; DTT: digit triplet test; FEV1: forced expiratory volume in one second; FVC: forced vital capacity; GWAS: genome-wide association study; GCSEs: general certificate of secondary education; HND: higher national diploma; HNC: higher national certificate; IV: instrumental variable; IQR: interquartile range; IVW: inverse-variance weighted; LLN: lower limit of normal; MR: Mendelian randomization; MR-PRESSO: Mendelian randomization pleiotropy residual sum and outlier; NVQ: national vocational qualification; OR: odds ratio; SRT: speech recognition threshold; SD: standard deviation; TDI: Townsend deprivation index.
