# Supplementary material for: Role of Lung Function, Chronic Obstructive Pulmonary Disease on Hearing Impairment: Evidence for Causal Effects and Clinical Implications
Source: Audiol Res. 2025 Jul 16;15(4):88. doi: 10.3390/audiolres15040088 (PMC12285983; doi:10.3390/audiolres15040088)
Supplement: Supplementary file 1 [file audiolres-15-00088-s001.zip › audiolres-3665285-supplementary.pdf]

# Supplementary material

**Role of lung function, chronic obstructive pulmonary disease on hearing impairment: evidence for causal effects and clinical implications**

Lanlai Yuan, Feipeng Cui, Ge Yin, Mengwen Shi, Nadida Aximu, Yaohua Tian, and Yu Sun

## Supplemental Text

### Supplemental Text 1

Sociodemographic variables included age, sex, educational level, occupation, and Townsend deprivation index (TDI). The TDI is a proxy for socioeconomic status, which was assigned to each participant based on their postcode at baseline. Higher values indicate lower socioeconomic status or higher levels of deprivation. For this study, the TDI was divided into quartiles. Lifestyle-related variables included smoking and the frequency of alcohol consumption. Body mass index was calculated as the participant's weight in kilograms divided by the square of their height in meters and was categorized into three groups: < 25 kg/m$^2$, 25−30 kg/m$^2$, and ≥ 30 kg/m$^2$. The history of chronic diseases, including diabetes and cardiovascular diseases, was self-reported through a touch-screen questionnaire. Noise exposure included occupational and musical noise.

### Supplemental Text 2

**Data sources for lung function**

Summary data for forced expiratory volume in one second (FEV1) (IEU GWAS ID: ukb-b-19657; unit in standard deviation) and forced vital capacity (FVC) (IEU GWAS ID: ukb-b-7953; unit in standard deviation) were extracted from the GWAS analyses of 421,986 individuals of European ancestry in the UK Biobank, which are publicly available in the Integrative Epidemiologic Unit GWAS database developed at

the MRC Integrative Epidemiology Unit at the University of Bristol (https://gwas.mrcieu.ac.uk/). The successfully genotyped samples from UK Biobank included 488,377 participants, which were assayed using two very similar genotyping arrays (the Affymetrix UK BiLEVE array and the Affymetrix UK Biobank axiom array) [1].The quality control of the data was performed before the GWAS [2].A linear mixed model was used in the GWAS to account for relatedness and population stratification, with adjustments for sex and genotyping array.

**Data sources for chronic obstructive pulmonary disease (COPD)**

Summary data for COPD were obtained from a GWAS meta-analysis of 25 studies from the UK Biobank and the International COPD Genetics Consortium (ICGC), with a total of 257,811 individuals of predominantly European ancestry (including 35,735 cases and 222,076 controls) [3]. On the basis of pre-bronchodilator spirometry, COPD was defined according to modified Global Initiative for Chronic Obstructive Lung Disease (GOLD) criteria for moderate to very severe airflow limitation. A fixed-effects meta-analysis was performed on all studies from the ICGC cohorts and the UK Biobank. Population substructure and cryptic relatedness were assessed using the intercept of the linkage disequilibrium score regression. This study identified 82 genome-wide significant ($P < 5 \times 10^{-8}$) loci (defined by using 2-Mb windows).

**Data sources for sensorineural hearing loss**

GWAS summary data for sensorineural hearing loss were downloaded from the FinnGen study (R9), with a total of 364,223 individuals (including 32,487 cases and 331,736 controls), which can be obtained from the official website (https://www.finngen.fi/en). The FinnGen study is a large-scale genomics initiative that has analyzed over 500,000 Finnish biobank samples and correlated genetic variation with health data to understand disease mechanisms and predispositions [4]. The project is a collaboration between research organizations and biobanks within Finland and international industry partners [4].

Quality control samples and chip genotype data processing were genotyped using Illumina (Illumina Inc., San Diego, CA, USA) and Affymetrix arrays (Thermo Fisher

Scientific, Santa Clara, CA, USA). The genome-wide association analysis was adjusted for sex, age, ten principal components, and genotyping batch. Sensorineural hearing loss (FinnGen phenocode: H8_HL_SEN_NAS) was defined using ICD-9 (3891) and ICD-10 (H90.3, H90.4, H90.5) codes.

**Selection of genetic instruments**

The genome-wide significance threshold ($P < 5 \times 10^{-8}$) was used to select exposure-associated single nucleotide polymorphisms (SNPs) as instrumental variables (IVs), removing SNPs in linkage disequilibrium ($r^2 < 0.001$ within 10,000 kilobase pairs). The strength of IVs was measured using the Cragg-Donald F-statistic, and any SNP with F > 10 was retained. The corresponding SNPs were then extracted from the GWAS summary data of the outcome. For missing SNPs, proxies in high linkage disequilibrium ($r^2 \geq 0.8$) with the original SNPs were used in Mendelian randomization (MR) analyses; these proxies were identified from the 1000 Genomes (European reference panel). SNPs without proxies were excluded. Harmonization was done to make sure that the effect alleles for exposure and outcome were the same.

**Supplemental Text 3**

**Cross-sectional analyses**

The Mean (standard deviation, SD) or the median (interquartile range, IQR) for continuous variables and the frequency (%) for categorical variables were used to describe the basic characteristics of the participants. Missing values (< 2.5%) were coded as "missing" for categorical covariates. Independent sample t-tests or Wilcoxon rank sum tests for continuous variables and Chi-squared tests for categorical variables were used to compare the differences between the normal hearing and hearing impairment groups.

The associations of lung function and COPD with the risk of hearing impairment were analyzed using multivariate logistic regression models. Two models were fitted: a crude model and an adjusted model, including age, sex, TDI, qualifications,

employment, smoking, drinking frequency, body mass index, diabetes, cardiovascular diseases, and noise exposure. Analyses were stratified by age (< 60 and ≥ 60 years) and sex (male and female) to investigate the consistency of associations across strata. The interactions of lung function and COPD with stratification variables were examined using a likelihood ratio test by adding a product term to the logistic regression models. In the sensitivity analysis, COPD defined as the FEV1/FVC ratio below 0.7 was used to repeat the analysis. Second, participants with a history of asthma were excluded to minimize exposure misclassification. Third, self-reported hearing difficulty was treated as a secondary outcome. Fourth, a restricted cubic spline model was used to explore the dose-response relationship between lung function and risk of hearing impairment, with three knots placed at the 10th, 50th, and 90th percentiles of the model. The minimum values of FEV1 and FVC were used as references, respectively.

**Two-sample MR**

In the MR analyses, Cochran's Q statistic was used to measure substantial heterogeneity among variant-specific causal estimates ($P$ < 0.05). Excessive heterogeneity indicates a violation of one or more of the assumptions in the MR study [5]. Additionally, radial, scatter, and funnel plots were used to explore heterogeneity and outliers. Outliers, if existing, were visually detected using radial plots and excluded before the primary MR analyses.

In the MR study, horizontal pleiotropy (violation of the exclusion restriction assumption) can severely bias the results [6]. Therefore, the MR-Egger intercept test was used to examine whether horizontal pleiotropy was present. An intercept with a significant deviation from zero indicates the presence of horizontal pleiotropy [7]. Furthermore, horizontal pleiotropic outliers were identified and corrected using the Mendelian randomization pleiotropy residual sum and outlier (MR-PRESSO) test, which consists of three tests: the MR-PRESSO global test, the MR-PRESSO outlier test, and the MR-PRESSO bias test [6]. A leave-one-out analysis was used to assess whether the estimation of causal effects was driven by any single SNP. The MR Steiger

test was also employed to evaluate the possibility of reverse association [8].

Consistent with the criteria for defining causal effects in previous MR studies [9, 10], when no evidence of heterogeneity or horizontal pleiotropy was found after excluding potential outliers, and sensitivity analyses showed results consistent in magnitude and direction with those of the inverse variance weighted (IVW) method, the existence of causality was suggested.

**IVW**

The IVW method is a weighted linear regression model proposed by Burgess et al [11]. It combines the Wald ratios (or ratio estimates) corresponding to multiple valid IVs, with the weight of each ratio being the inverse of the variance of the association between a SNP and outcome [11]. In the IVW method, it is assumed that there is no horizontal pleiotropy, and the intercept is constrained to be equal to zero [12]. When there is no pleiotropy in IVs, the IVW method provides an unbiased estimate of causal effects, which is much more accurate and has greater power to reject the null hypothesis of causality [7].

**MR-Egger regression**

Similar to the IVW method, MR Egger regression is a weighted linear regression model, but the intercept is not constrained [7]. The estimated intercept in Egger regression can be interpreted as an assessment of the average pleiotropic effect among genetic variants, with a nonzero intercept term indicating an overall directional pleiotropy [7]. It is assumed in MR-Egger regression that the association of genetic variants with exposure is independent of the direct effect of genetic variants on the outcome (i.e., the InSIDE assumption), which is a weaker assumption than the assumption that all genetic variants are valid IVs [7, 13]. Under the InSIDE assumption, MR Egger regression provides valid tests for directional (unbalanced) pleiotropy and the causal null hypothesis, with the slope estimate being a consistent estimate of the true causal effects [7]. If the InSIDE assumption is violated, MR Egger regression will not provide a consistent estimate of causal effects, but it is still a more robust inference method compared to the standard method that relies on stronger hypotheses without

pleiotropy [7]. However, due to the power penalty, the MR Egger regression is less accurate than the estimates obtained by other methods [14]. Therefore, MR Egger regression is an important sensitivity analysis tool in MR [7].

**Weighted median**

The weighted median method combines data from multiple genetic variants into one causal estimate [15]. Even if up to half of the weight in the analysis is derived from invalid genetic IVs, the weighted median method can yield a consistent estimate of causal effects [15].

**Reference**


1. Bycroft C, Freeman C, Petkova D, et al. The UK Biobank resource with deep phenotyping and genomic data. *Nature* 2018: 562(7726): 203-209.
2. MRC IEU UK Biobank GWAS pipeline, version 2, 18/01/2019. cited Available from:https://data.bris.ac.uk/datasets/pnoat8cxo0u52p6ynfaekeigi/MRC%20IEU%20UK%20Biobank%20GWAS%20pipeline%20version%202.pdf
3. Sakornsakolpat P, Prokopenko D, Lamontagne M, et al. Genetic landscape of chronic obstructive pulmonary disease identifies heterogeneous cell-type and phenotype associations. *Nature genetics* 2019: 51(3): 494-505.
4. Kurki MI, Karjalainen J, Palta P, et al. FinnGen provides genetic insights from a well-phenotyped isolated population. *Nature* 2023: 613(7944): 508-518.
5. Bowden J, Spiller W, Del Greco MF, et al. Improving the visualization, interpretation and analysis of two-sample summary data Mendelian randomization via the Radial plot and Radial regression. *International journal of epidemiology* 2018: 47(4): 1264-1278.
6. Verbanck M, Chen CY, Neale B, Do R. Detection of widespread horizontal pleiotropy in causal relationships inferred from Mendelian randomization between complex traits and diseases. *Nature genetics* 2018: 50(5): 693-698.
7. Bowden J, Davey Smith G, Burgess S. Mendelian randomization with invalid instruments: effect estimation and bias detection through Egger regression. *International journal of epidemiology* 2015: 44(2): 512-525.
8. Hemani G, Tilling K, Davey Smith G. Orienting the causal relationship between imprecisely measured traits using GWAS summary data. *PLoS genetics* 2017: 13(11): e1007081.
9. Au Yeung SL, Borges MC, Lawlor DA. Association of Genetic Instrumental Variables for Lung Function on Coronary Artery Disease Risk: A 2-Sample Mendelian Randomization Study. *Circulation Genomic and precision medicine* 2018: 11(4): e001952.
10. Guo B, Wang C, Zhu Y, et al. Causal associations of brain structure with bone mineral density: a large-scale genetic correlation study. *Bone research* 2023: 11(1):

37.
11. Burgess S, Butterworth A, Thompson SG. Mendelian randomization analysis with multiple genetic variants using summarized data. *Genetic epidemiology* 2013: 37(7): 658-665.
12. Bowden J, Del Greco MF, Minelli C, Davey Smith G, Sheehan N, Thompson J. A framework for the investigation of pleiotropy in two-sample summary data Mendelian randomization. *Statistics in medicine* 2017: 36(11): 1783-1802.
13. Skrivankova VW, Richmond RC, Woolf BAR, et al. Strengthening the reporting of observational studies in epidemiology using mendelian randomisation (STROBE-MR): explanation and elaboration. *BMJ* 2021: 375: n2233.
14. Davies NM, Holmes MV, Davey Smith G. Reading Mendelian randomisation studies: a guide, glossary, and checklist for clinicians. *BMJ* 2018: 362: k601.
15. Bowden J, Davey Smith G, Haycock PC, Burgess S. Consistent Estimation in Mendelian Randomization with Some Invalid Instruments Using a Weighted Median Estimator. *Genetic epidemiology* 2016: 40(4): 304-314.

**Figures**

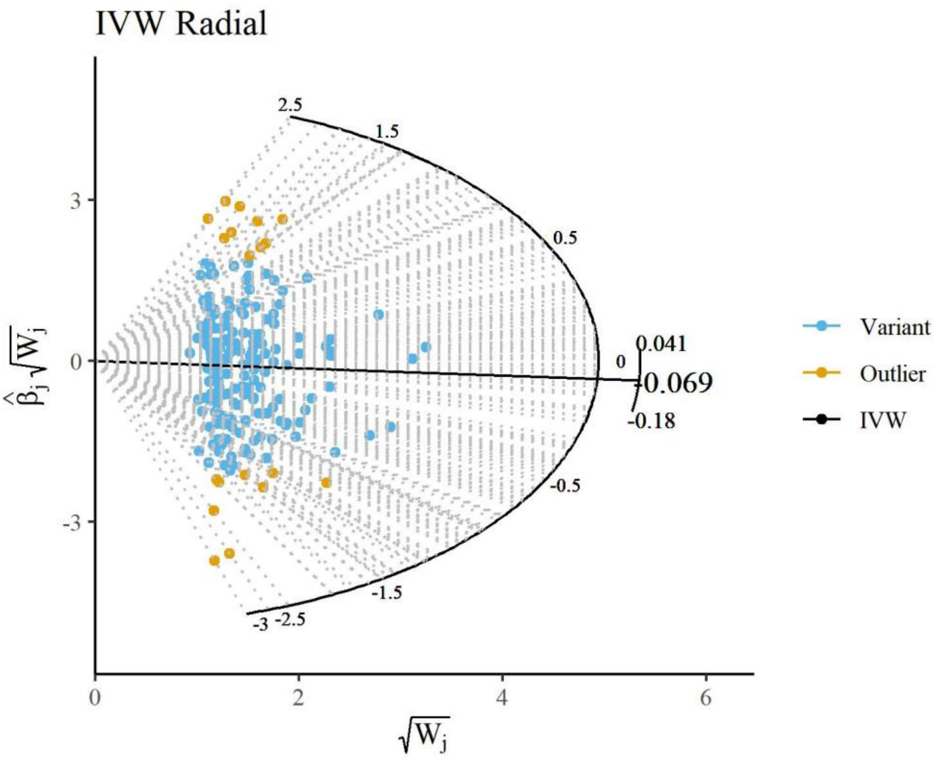


**Figure S1.** A radial plot of the association between FEV1 and sensorineural hearing loss.

FEV1, forced expiratory volume in one second; IVW, inverse variance weighted.

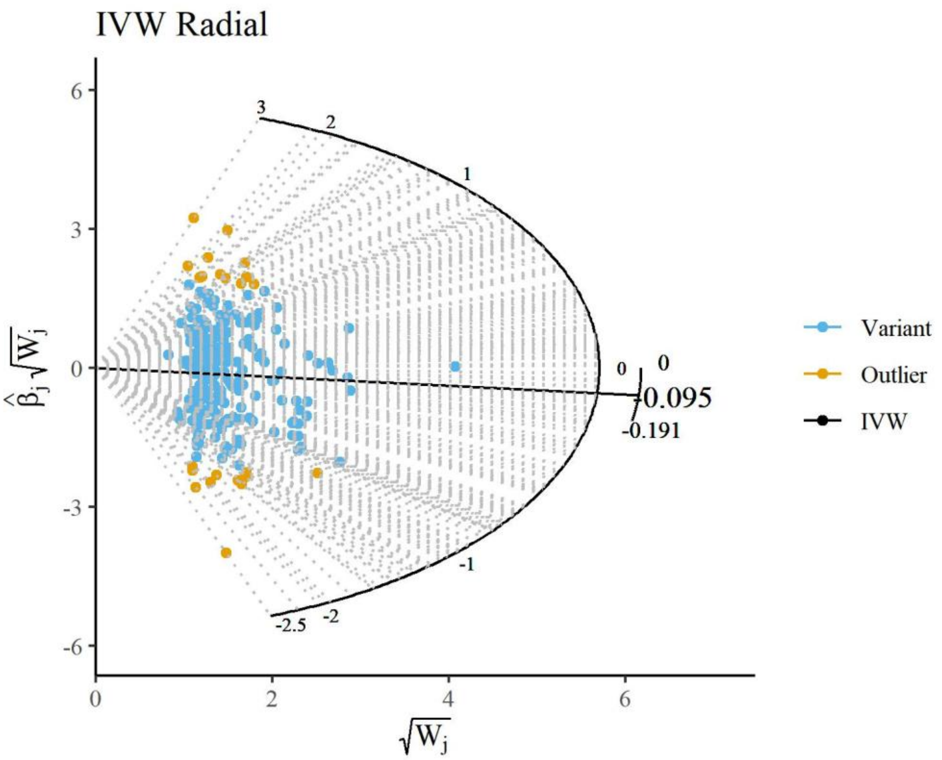


**Figure S2.** A radial plot of the association between FVC and sensorineural hearing loss.
FVC, forced vital capacity; IVW, inverse variance weighted.

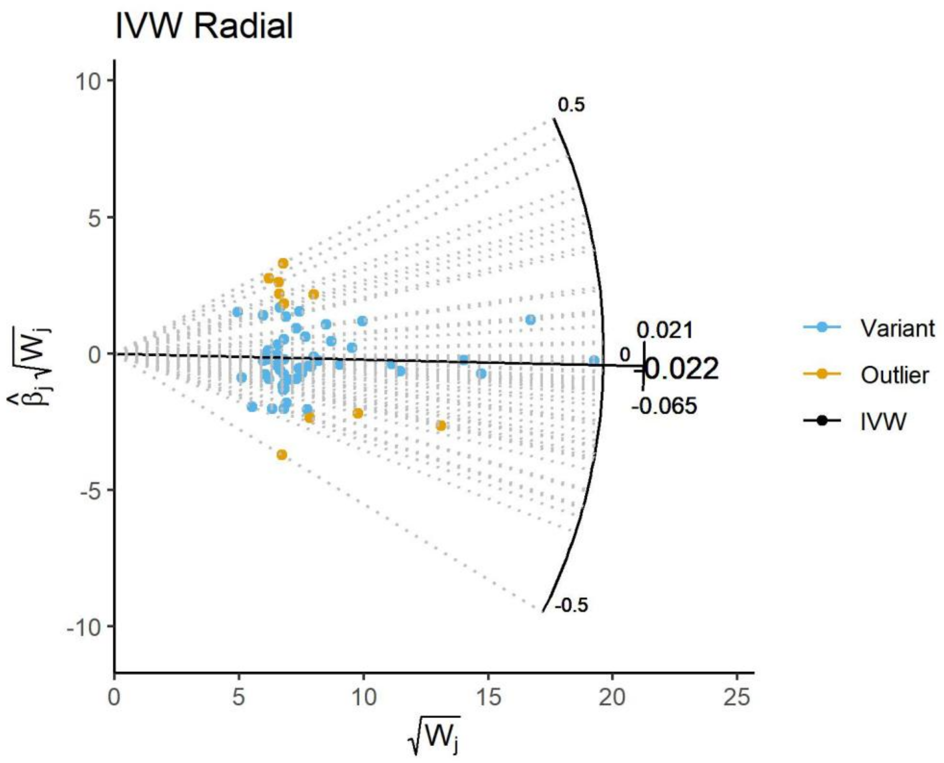


**Figure S3.** A radial plot of the association between COPD and sensorineural hearing loss.

COPD, chronic obstructive pulmonary disease; IVW, inverse variance weighted.

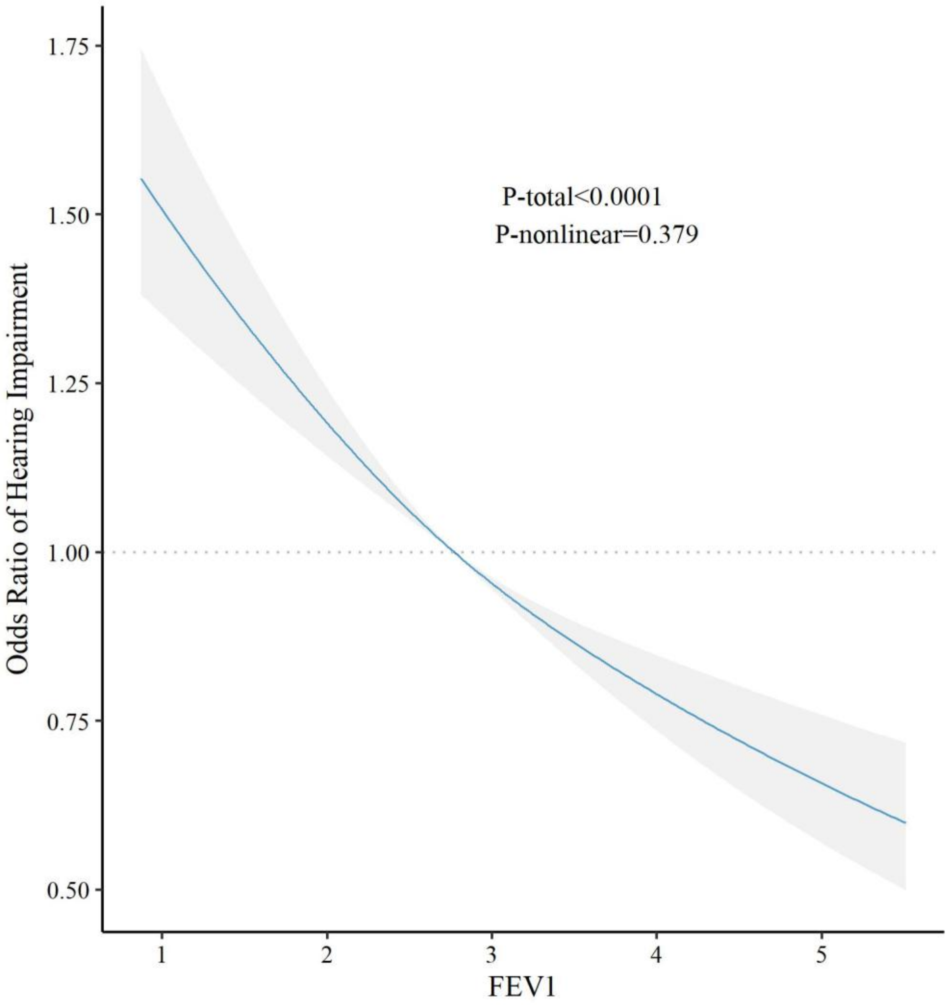


**Figure S4.** Dose-response curve of FEV1 with odds of hearing impairment using restricted cubic spline model.

The three Harrel knots of the restricted cubic spline model were placed at the 10th, 50th, and 90th percentiles of the FEV1.

FEV1, forced expiratory volume in one second.

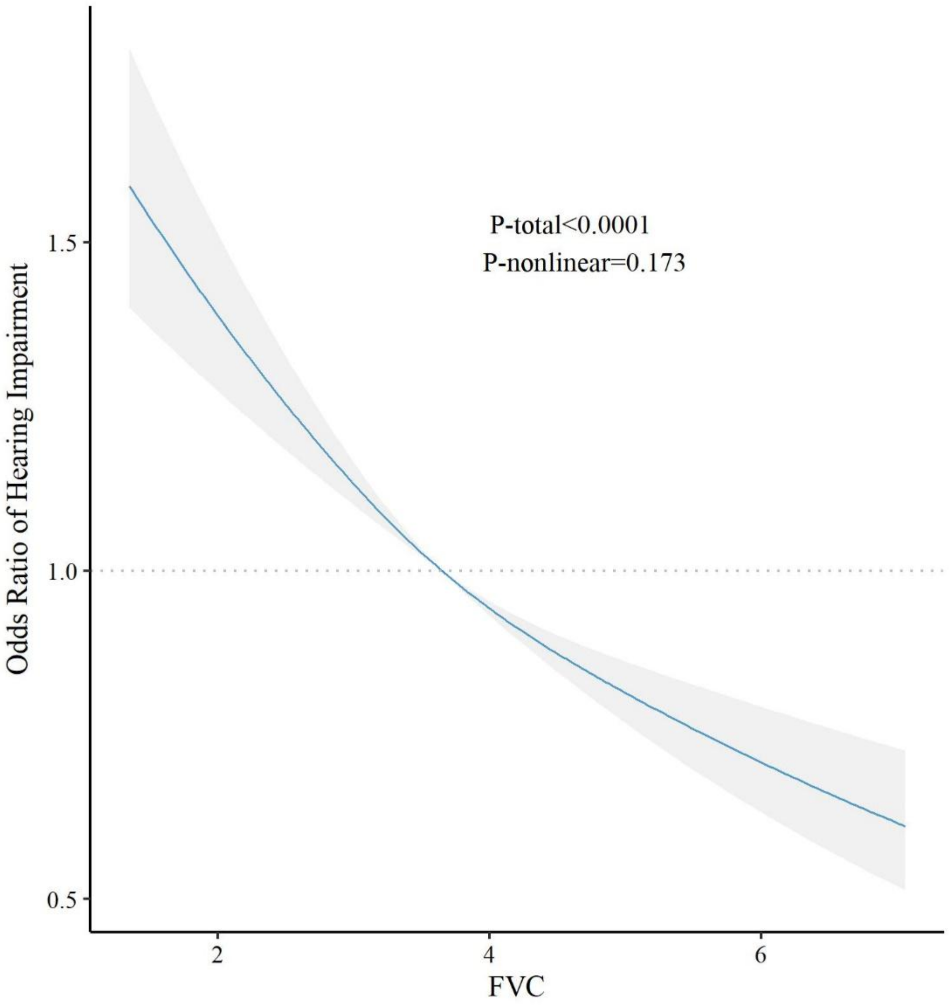


**Figure S5.** Dose-response curve of FVC with odds of hearing impairment using restricted cubic spline model.

The three Harrel knots of the restricted cubic spline model were placed at the 10th, 50th, and 90th percentiles of the FVC.

FVC, forced vital capacity.

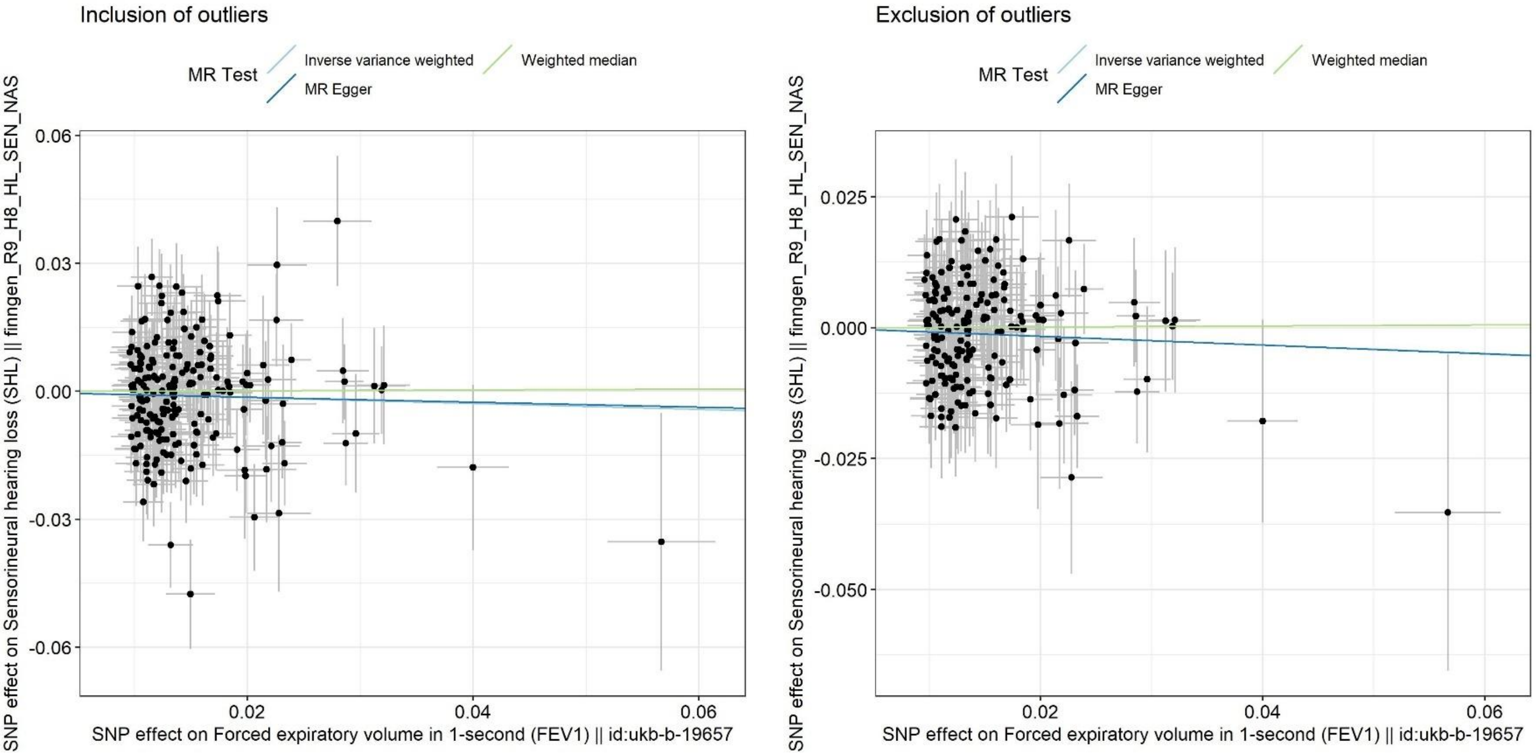


**Figure S6.** Scatter plot of SNP-effect on FEV1 (x-axis) and SNP-effect on sensorineural hearing loss (y-axis).

MR, Mendelian randomization; SNP, single nucleotide polymorphism; FEV1, forced expiratory volume in one second.

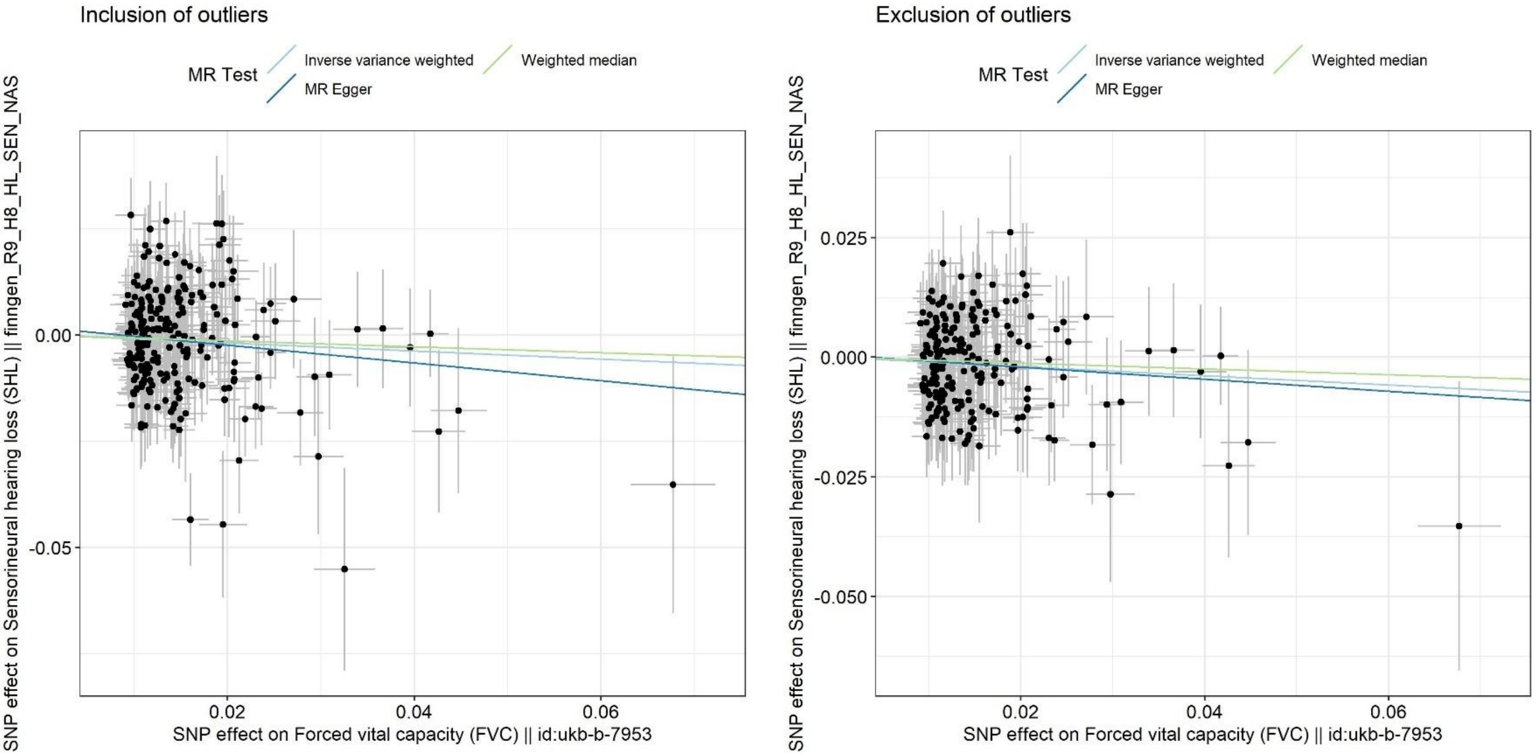


**Figure S7.** Scatter plot of SNP-effect on FVC (x-axis) and SNP-effect on sensorineural hearing loss (y-axis).

MR, Mendelian randomization; SNP, single nucleotide polymorphism; FVC, forced vital capacity.

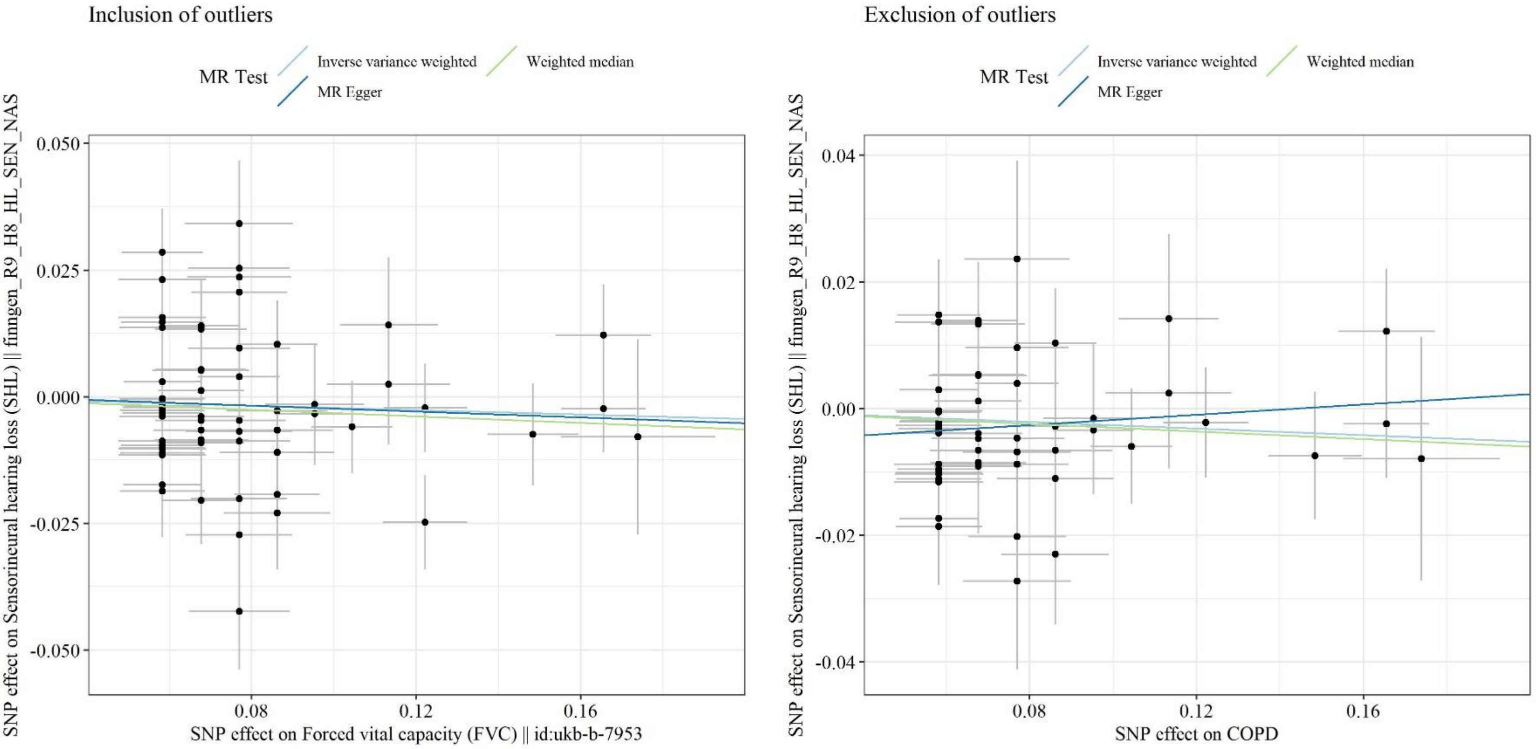


**Figure S8.** Scatter plot of SNP-effect on COPD (x-axis) and SNP-effect on sensorineural hearing loss (y-axis).

MR, Mendelian randomization; SNP, single nucleotide polymorphism; COPD, chronic obstructive pulmonary disease.

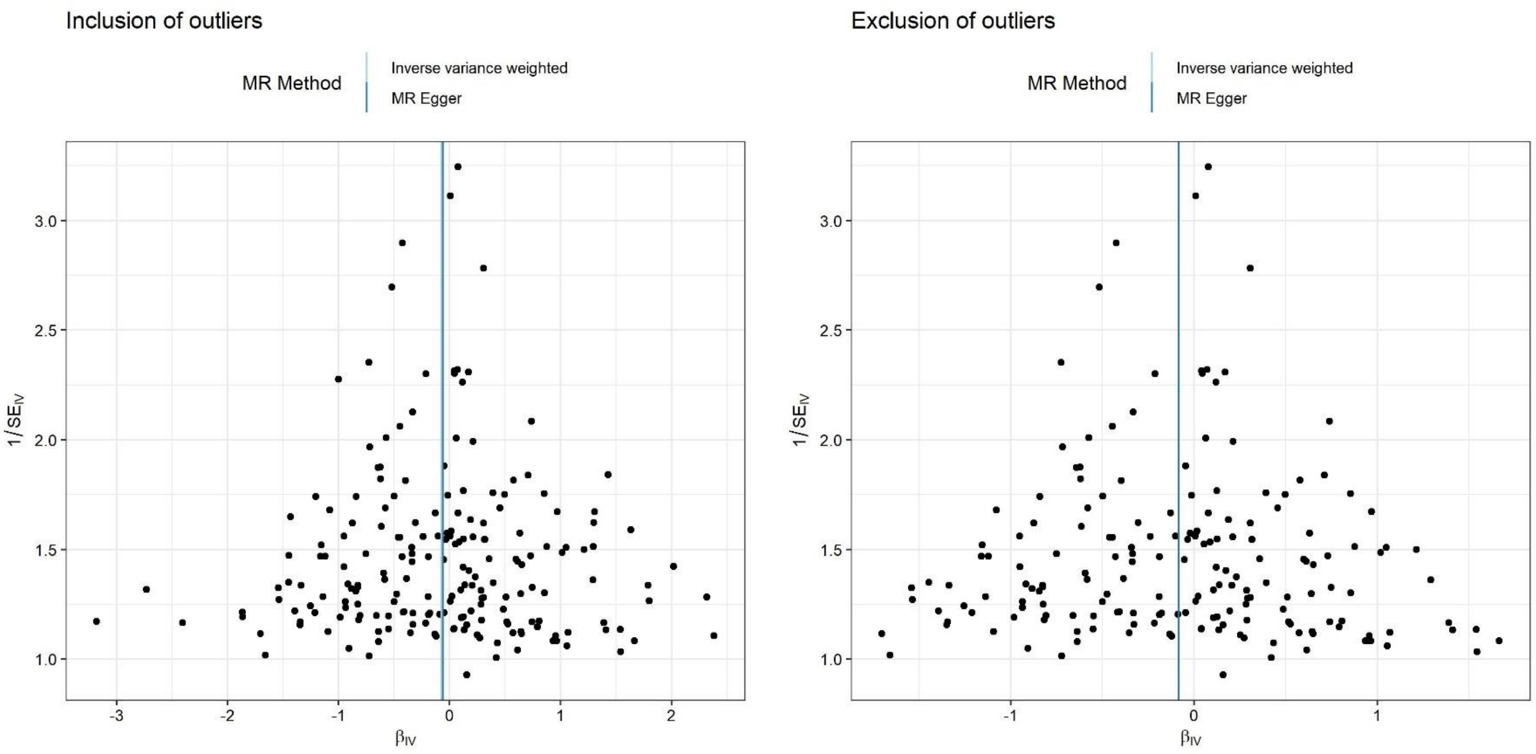


**Figure S9.** Funnel plot of the heterogeneity of the causal effect of FEV1 on sensorineural hearing loss.

MR, Mendelian randomization; FEV1, forced expiratory volume in one second.

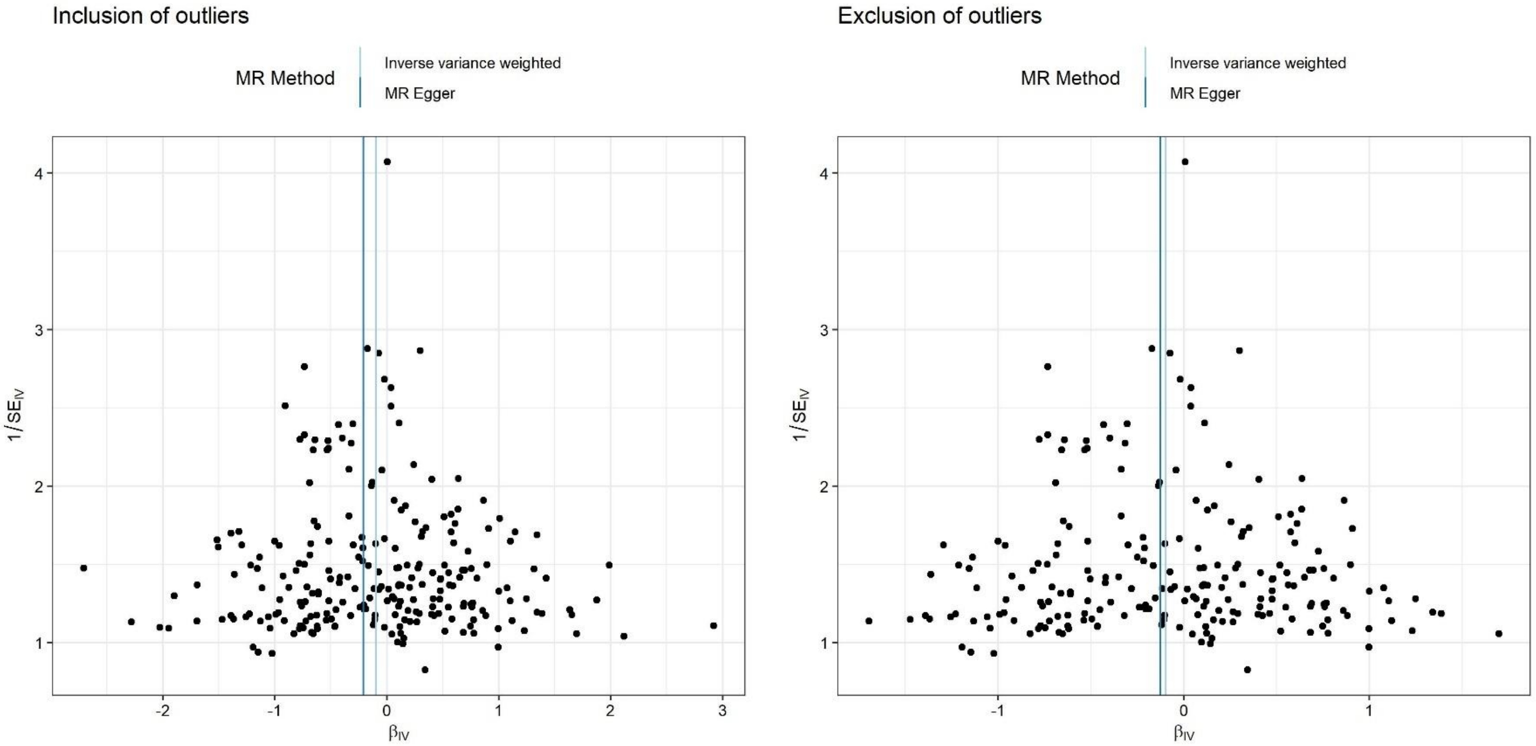


**Figure S10.** Funnel plot of the heterogeneity of the causal effect of FVC on sensorineural hearing loss.

MR, Mendelian randomization; FVC, forced vital capacity.

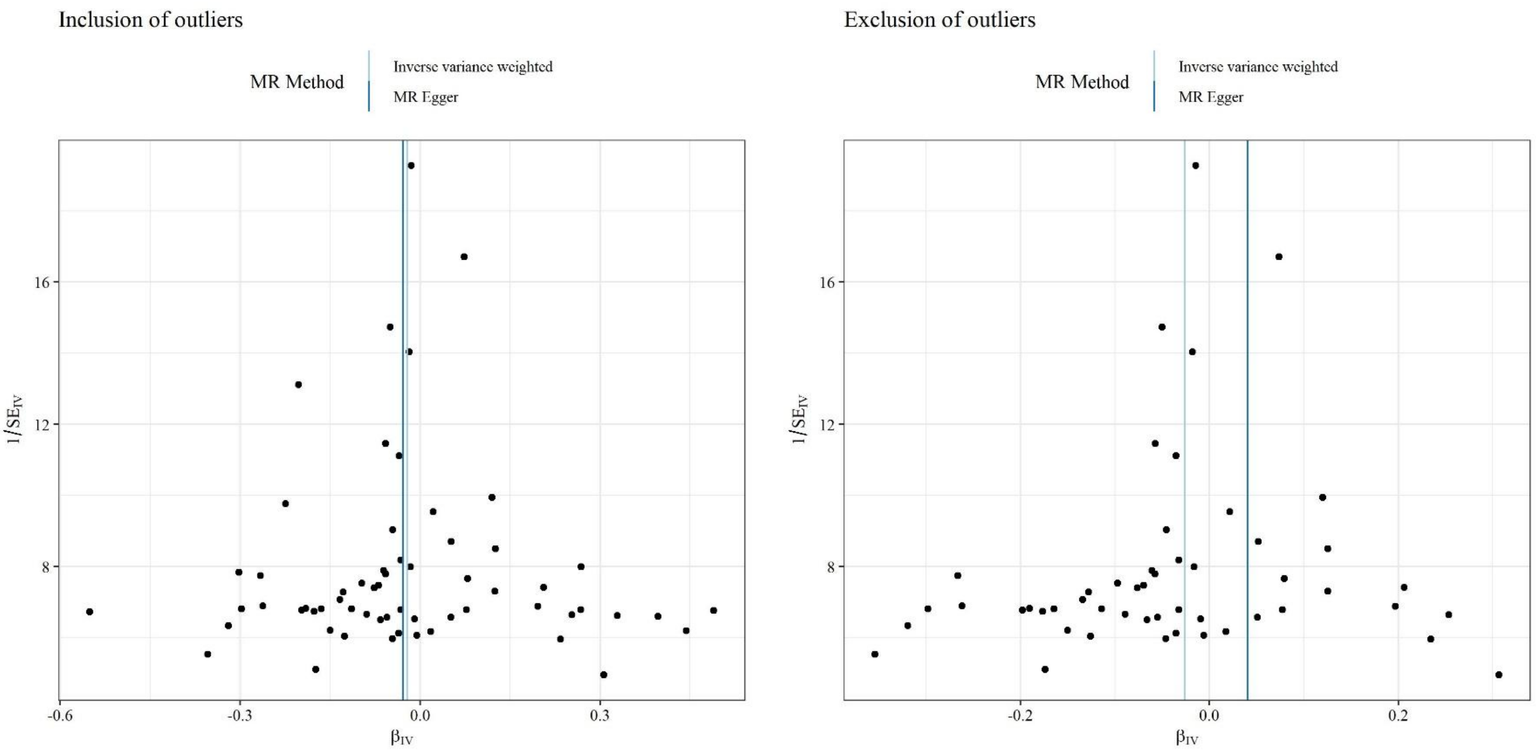


**Figure S11.** Funnel plot of the heterogeneity of the causal effect of COPD on sensorineural hearing loss.

MR, Mendelian randomization; COPD, chronic obstructive pulmonary disease.

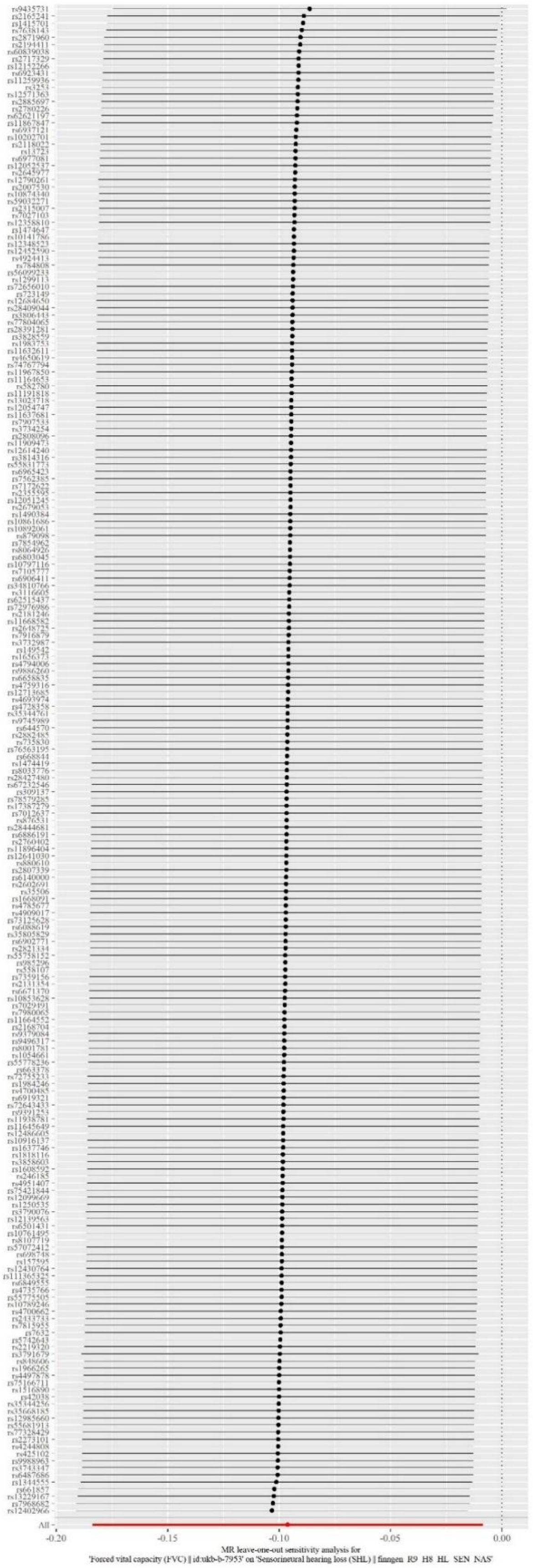


**Figure S12.** Leave-one-out analysis of the causal effect of FVC on sensorineural hearing loss after excluding radial IVW outliers.

FVC, forced vital capacity; IVW, inverse variance weighted.

## Tables

**Table S1.** Information of the genome wide association studies included in this study.

| Exposure | Data source | Sample size (Number of cases) | Ancestry | Unit | IEU GWAS ID or FinnGen phenocode |
|---|---|---|---|---|---|
| FEV1 | UK Biobank | 421,986 | European | SD | ukb-b-19657 |
| FVC | UK Biobank | 421,986 | European | SD | ukb-b-7953 |
| COPD | PMID: 30804561 (UK Biobank and International COPD Genetics Consortium) | 257,811 (35,735) | Mixed ancestry (predominantly European) | Log odds | NA |
| **Outcome** | | | | | |
| Sensorineural hearing loss | FinnGen (R9) | 364,223 (32,487) | European | Log odds | H8_HL_SEN_NAS |

IEU, Integrative Epidemiologic Unit; GWAS, genome-wide association study; FEV1, forced expiratory volume in one second; FVC, forced vital capacity; COPD, chronic obstructive pulmonary disease; SD, standard deviation.

**Table S2.** Genetic instruments for FEV1.

| SNP | Effect allele | Other allele | Effect allele frequency | Beta | Standard error | Sample size | P value | Exposure | R2 | F statistic | Outliers (Radial IVW) |
|---|---|---|---|---|---|---|---|---|---|---|---|
| rs10107584 | T | C | 0.611245 | 0.00982184 | 0.00179714 | 421986 | 4.60E-08 | FEV1 | 3.40E-05 | 14.347944 | Variant |
| rs10141786 | G | A | 0.596493 | -0.0121024 | 0.00177709 | 421986 | 9.70E-12 | FEV1 | 5.30E-05 | 22.366337 | Variant |
| rs10167980 | G | A | 0.734288 | -0.0111231 | 0.00196218 | 421986 | 1.40E-08 | FEV1 | 3.00E-05 | 12.6599 | Variant |
| rs10490551 | A | T | 0.276305 | 0.013707 | 0.00193897 | 421986 | 1.60E-12 | FEV1 | 4.70E-05 | 19.83418 | Variant |
| rs1053049 | T | C | 0.763272 | 0.0216134 | 0.00204457 | 421986 | 4.10E-26 | FEV1 | 9.60E-05 | 40.514353 | Variant |
| rs10789246 | C | T | 0.301089 | -0.011118 | 0.00188847 | 421986 | 3.90E-09 | FEV1 | 3.50E-05 | 14.769957 | Variant |
| rs10797116 | C | T | 0.542079 | -0.0126837 | 0.00174658 | 421986 | 3.80E-13 | FEV1 | 6.20E-05 | 26.16463 | Variant |
| rs10851839 | A | T | 0.668554 | 0.0103302 | 0.00184696 | 421986 | 2.20E-08 | FEV1 | 3.30E-05 | 13.925932 | Outlier |
| rs10883928 | T | C | 0.359436 | -0.0135899 | 0.00180959 | 421986 | 5.90E-14 | FEV1 | 6.20E-05 | 26.16463 | Variant |
| rs10911335 | C | T | 0.21106 | 0.0160149 | 0.00212544 | 421986 | 4.90E-14 | FEV1 | 4.50E-05 | 18.990135 | Variant |
| rs10965947 | T | C | 0.458692 | 0.0129574 | 0.00174086 | 421986 | 9.80E-14 | FEV1 | 6.50E-05 | 27.430743 | Variant |
| rs11049381 | A | G | 0.564116 | 0.0134548 | 0.00175227 | 421986 | 1.60E-14 | FEV1 | 6.90E-05 | 29.118905 | Variant |
| rs11134789 | A | C | 0.341728 | -0.01435 | 0.00182876 | 421986 | 4.30E-15 | FEV1 | 6.60E-05 | 27.852782 | Outlier |
| rs11166592 | T | G | 0.577472 | 0.0125753 | 0.00176215 | 421986 | 9.60E-13 | FEV1 | 5.90E-05 | 24.898525 | Variant |
| rs11259936 | C | A | 0.52286 | 0.023076 | 0.00173772 | 421986 | 3.00E-40 | FEV1 | 0.000209 | 88.213093 | Variant |
| rs112723784 | C | T | 0.27797 | 0.0105929 | 0.00194023 | 421986 | 4.80E-08 | FEV1 | 2.80E-05 | 11.815883 | Variant |
| rs11559147 | G | C | 0.709183 | 0.0122536 | 0.00192261 | 421986 | 1.80E-10 | FEV1 | 4.00E-05 | 16.880035 | Variant |
| rs11621587 | C | G | 0.183978 | 0.0206492 | 0.00223832 | 421986 | 2.80E-20 | FEV1 | 6.10E-05 | 25.742594 | Outlier |
| rs11623570 | A | C | 0.232947 | -0.0117 | 0.00205404 | 421986 | 1.20E-08 | FEV1 | 2.70E-05 | 11.393876 | Variant |
| rs11641308 | C | T | 0.654691 | -0.0107516 | 0.00184605 | 421986 | 5.70E-09 | FEV1 | 3.60E-05 | 15.191971 | Variant |
| rs11674239 | G | C | 0.266298 | -0.0110821 | 0.00196738 | 421986 | 1.80E-08 | FEV1 | 2.90E-05 | 12.237891 | Variant |
| rs11689727 | A | C | 0.331368 | -0.0111838 | 0.00184432 | 421986 | 1.30E-09 | FEV1 | 3.90E-05 | 16.458018 | Outlier |
| rs11707746 | C | T | 0.286941 | -0.0118845 | 0.00192893 | 421986 | 7.20E-10 | FEV1 | 3.70E-05 | 15.613986 | Variant |
| rs1187115 | A | C | 0.830007 | -0.0284605 | 0.00230876 | 421986 | 6.50E-35 | FEV1 | 0.000102 | 43.046759 | Variant |
| rs11877758 | G | T | 0.3121 | -0.0117186 | 0.00188255 | 421986 | 4.80E-10 | FEV1 | 3.90E-05 | 16.458018 | Outlier |
| rs11909473 | G | A | 0.28953 | 0.011076 | 0.00192519 | 421986 | 8.80E-09 | FEV1 | 3.20E-05 | 13.50392 | Variant |
| rs11938781 | C | T | 0.165403 | -0.032099 | 0.0023426 | 421986 | 9.80E-43 | FEV1 | 0.000123 | 51.910417 | Variant |
| rs12028838 | T | G | 0.447808 | -0.00959229 | 0.00174239 | 421986 | 3.70E-08 | FEV1 | 3.60E-05 | 15.191971 | Variant |
| rs12152266 | T | C | 0.27447 | 0.0141313 | 0.00194446 | 421986 | 3.70E-13 | FEV1 | 5.00E-05 | 21.100255 | Variant |
| rs12358810 | A | G | 0.392273 | -0.0114193 | 0.00178594 | 421986 | 1.60E-10 | FEV1 | 4.60E-05 | 19.412157 | Variant |
| rs12413039 | G | C | 0.267488 | 0.0142023 | 0.00195704 | 421986 | 4.00E-13 | FEV1 | 4.90E-05 | 20.678229 | Outlier |
| rs12571363 | A | C | 0.10664 | -0.0228143 | 0.00281378 | 421986 | 5.10E-16 | FEV1 | 3.00E-05 | 12.6599 | Variant |
| rs12641030 | G | A | 0.467529 | -0.0131533 | 0.00173853 | 421986 | 3.90E-14 | FEV1 | 6.80E-05 | 28.696863 | Variant |
| rs12645165 | C | T | 0.364275 | 0.0135283 | 0.00180117 | 421986 | 5.90E-14 | FEV1 | 6.20E-05 | 26.16463 | Variant |
| rs12684650 | T | C | 0.316579 | -0.0165789 | 0.0018661 | 421986 | 6.40E-19 | FEV1 | 8.10E-05 | 34.183473 | Variant |
| rs12698403 | A | G | 0.439685 | -0.0139202 | 0.00175386 | 421986 | 2.10E-15 | FEV1 | 7.40E-05 | 31.229127 | Variant |
| rs12790261 | A | C | 0.082405 | -0.0399934 | 0.00316131 | 421986 | 1.10E-36 | FEV1 | 5.70E-05 | 24.054459 | Variant |
| rs12894780 | C | T | 0.126445 | -0.0226382 | 0.00262602 | 421986 | 6.70E-18 | FEV1 | 3.90E-05 | 16.458018 | Outlier |
| rs12917612 | A | C | 0.232311 | -0.012019 | 0.00205725 | 421986 | 5.10E-09 | FEV1 | 2.90E-05 | 12.237891 | Variant |
| rs12985660 | C | T | 0.475562 | -0.0098092 | 0.00174527 | 421986 | 1.90E-08 | FEV1 | 3.70E-05 | 15.613986 | Variant |
| rs13044144 | C | G | 0.262827 | 0.0158381 | 0.00197778 | 421986 | 1.20E-15 | FEV1 | 5.90E-05 | 24.898525 | Variant |
| rs13101469 | T | A | 0.196758 | -0.0152995 | 0.00218825 | 421986 | 2.70E-12 | FEV1 | 3.70E-05 | 15.613986 | Variant |

| | | | | | | | | | | | |
|---|---|---|---|---|---|---|---|---|---|---|---|
| rs13178887 | C | T | 0.386603 | -0.0103386 | 0.00177913 | 421986 | 6.20E-09 | FEV1 | 3.80E-05 | 16.036001 | Variant |
| rs13229167 | A | G | 0.271146 | -0.018447 | 0.00195743 | 421986 | 4.30E-21 | FEV1 | 8.30E-05 | 35.027579 | Variant |
| rs13429514 | A | G | 0.23795 | 0.0136376 | 0.00203576 | 421986 | 2.10E-11 | FEV1 | 3.90E-05 | 16.458018 | Variant |
| rs13433809 | G | A | 0.439229 | -0.0128272 | 0.00174729 | 421986 | 2.10E-13 | FEV1 | 6.30E-05 | 26.586667 | Variant |
| rs1344674 | G | A | 0.446057 | 0.0139325 | 0.0017434 | 421986 | 1.30E-15 | FEV1 | 7.50E-05 | 31.651174 | Variant |
| rs1390403 | A | C | 0.808334 | 0.0149666 | 0.00220795 | 421986 | 1.20E-11 | FEV1 | 3.40E-05 | 14.347944 | Variant |
| rs1415701 | A | G | 0.267657 | -0.0233013 | 0.00198294 | 421986 | 7.00E-32 | FEV1 | 0.000128 | 54.020867 | Variant |
| rs1490384 | T | C | 0.500495 | 0.0196914 | 0.00173383 | 421986 | 6.80E-30 | FEV1 | 0.000153 | 64.573432 | Variant |
| rs1516890 | A | G | 0.469749 | -0.0130475 | 0.00174914 | 421986 | 8.70E-14 | FEV1 | 6.60E-05 | 27.852782 | Variant |
| rs157580 | A | G | 0.605625 | 0.0102918 | 0.00177323 | 421986 | 6.50E-09 | FEV1 | 3.80E-05 | 16.036001 | Variant |
| rs1668091 | C | T | 0.31711 | 0.0108304 | 0.00187606 | 421986 | 7.80E-09 | FEV1 | 3.40E-05 | 14.347944 | Variant |
| rs1679910 | A | G | 0.437215 | 0.0097413 | 0.001762 | 421986 | 3.20E-08 | FEV1 | 3.60E-05 | 15.191971 | Variant |
| rs17178377 | A | G | 0.484564 | -0.0156306 | 0.00172788 | 421986 | 1.50E-19 | FEV1 | 9.70E-05 | 40.936419 | Variant |
| rs1829935 | G | A | 0.250856 | -0.0184902 | 0.00200267 | 421986 | 2.60E-20 | FEV1 | 7.60E-05 | 32.073222 | Variant |
| rs1867264 | T | A | 0.384943 | -0.0107832 | 0.00178929 | 421986 | 1.70E-09 | FEV1 | 4.10E-05 | 17.302053 | Outlier |
| rs1966265 | A | G | 0.245129 | 0.0135248 | 0.00201382 | 421986 | 1.90E-11 | FEV1 | 4.00E-05 | 16.880035 | Variant |
| rs1983753 | A | G | 0.851273 | 0.0197842 | 0.00245174 | 421986 | 7.10E-16 | FEV1 | 3.90E-05 | 16.458018 | Variant |
| rs2005172 | C | A | 0.64023 | 0.0198386 | 0.00183045 | 421986 | 2.30E-27 | FEV1 | 0.000128 | 54.020867 | Outlier |
| rs2010492 | G | A | 0.665231 | -0.0139699 | 0.0018418 | 421986 | 3.30E-14 | FEV1 | 6.10E-05 | 25.742594 | Variant |
| rs2099930 | A | G | 0.34083 | 0.0110552 | 0.00189199 | 421986 | 5.10E-09 | FEV1 | 3.60E-05 | 15.191971 | Variant |
| rs2118022 | C | T | 0.360318 | -0.0101606 | 0.00181403 | 421986 | 2.10E-08 | FEV1 | 3.40E-05 | 14.347944 | Variant |
| rs2194411 | A | G | 0.128337 | 0.0217026 | 0.00262547 | 421986 | 1.40E-16 | FEV1 | 3.60E-05 | 15.191971 | Variant |
| rs2236610 | G | C | 0.199727 | -0.0125089 | 0.0021751 | 421986 | 8.90E-09 | FEV1 | 2.50E-05 | 10.549864 | Variant |
| rs225646 | A | G | 0.757234 | 0.0167576 | 0.0020231 | 421986 | 1.20E-16 | FEV1 | 6.00E-05 | 25.320559 | Variant |
| rs2292839 | A | T | 0.219592 | 0.0118305 | 0.00209518 | 421986 | 1.60E-08 | FEV1 | 2.60E-05 | 10.971869 | Variant |
| rs2327790 | G | A | 0.702446 | 0.0105458 | 0.00190444 | 421986 | 3.10E-08 | FEV1 | 3.00E-05 | 12.6599 | Variant |
| rs2345443 | G | A | 0.690225 | -0.0119531 | 0.00187776 | 421986 | 1.90E-10 | FEV1 | 4.10E-05 | 17.302053 | Variant |
| rs2412981 | C | G | 0.908102 | -0.0279897 | 0.00300763 | 421986 | 1.30E-20 | FEV1 | 3.40E-05 | 14.347944 | Outlier |
| rs2415128 | C | T | 0.737026 | -0.0132057 | 0.00197595 | 421986 | 2.30E-11 | FEV1 | 4.10E-05 | 17.302053 | Outlier |
| rs2450444 | A | G | 0.348866 | 0.0134235 | 0.00181913 | 421986 | 1.60E-13 | FEV1 | 5.90E-05 | 24.898525 | Variant |
| rs2544535 | C | T | 0.513332 | 0.0135193 | 0.0017354 | 421986 | 6.70E-15 | FEV1 | 7.20E-05 | 30.385036 | Variant |
| rs2555009 | G | A | 0.534585 | 0.00999505 | 0.00173809 | 421986 | 8.90E-09 | FEV1 | 3.90E-05 | 16.458018 | Variant |
| rs2579762 | C | A | 0.471399 | -0.0151761 | 0.00173678 | 421986 | 2.40E-18 | FEV1 | 9.00E-05 | 37.981978 | Variant |
| rs2602691 | G | A | 0.449662 | -0.00977323 | 0.00175814 | 421986 | 2.70E-08 | FEV1 | 3.60E-05 | 15.191971 | Variant |
| rs262827 | T | A | 0.336148 | 0.0142653 | 0.00183602 | 421986 | 7.90E-15 | FEV1 | 6.40E-05 | 27.008705 | Variant |
| rs2645977 | A | G | 0.357445 | 0.0100995 | 0.00180964 | 421986 | 2.40E-08 | FEV1 | 3.40E-05 | 14.347944 | Variant |
| rs264770 | C | T | 0.671374 | 0.0105473 | 0.00184573 | 421986 | 1.10E-08 | FEV1 | 3.40E-05 | 14.347944 | Variant |
| rs2678408 | C | T | 0.46299 | -0.0133678 | 0.00174456 | 421986 | 1.80E-14 | FEV1 | 6.90E-05 | 29.118905 | Variant |
| rs2717329 | A | G | 0.294673 | 0.0160165 | 0.00193769 | 421986 | 1.40E-16 | FEV1 | 6.70E-05 | 28.274822 | Variant |
| rs278064 | C | T | 0.376633 | -0.0107284 | 0.00179279 | 421986 | 2.20E-09 | FEV1 | 4.00E-05 | 16.880035 | Variant |
| rs2806356 | C | T | 0.185674 | -0.019879 | 0.00222854 | 421986 | 4.70E-19 | FEV1 | 5.70E-05 | 24.054459 | Variant |
| rs2808096 | G | A | 0.21446 | 0.0124524 | 0.00211416 | 421986 | 3.90E-09 | FEV1 | 2.80E-05 | 11.815883 | Variant |
| rs28409044 | A | C | 0.333596 | 0.0111861 | 0.00184115 | 421986 | 1.20E-09 | FEV1 | 3.90E-05 | 16.458018 | Variant |
| rs28427480 | C | A | 0.093765 | 0.0231464 | 0.00298781 | 421986 | 9.40E-15 | FEV1 | 2.40E-05 | 10.127859 | Variant |
| rs2885697 | T | G | 0.665178 | -0.0169491 | 0.00183524 | 421986 | 2.60E-20 | FEV1 | 9.00E-05 | 37.981978 | Variant |
| rs2971040 | C | T | 0.518228 | 0.00967267 | 0.00173783 | 421986 | 2.60E-08 | FEV1 | 3.70E-05 | 15.613986 | Variant |
| rs30232 | A | G | 0.582657 | 0.011432 | 0.00175946 | 421986 | 8.20E-11 | FEV1 | 4.90E-05 | 20.678229 | Variant |

| | | | | | | | | | | | | |
|---|---|---|---|---|---|---|---|---|---|---|---|---|
| rs3091552 | G | C | 0.735538 | -0.0154813 | 0.00201796 | 421986 | 1.70E-14 | FEV1 | 5.40E-05 | 22.788367 | Variant |
| rs3253 | T | C | 0.314156 | 0.0110971 | 0.00186964 | 421986 | 2.90E-09 | FEV1 | 3.60E-05 | 15.191971 | Variant |
| rs34587839 | A | G | 0.154712 | -0.0174389 | 0.00240416 | 421986 | 4.10E-13 | FEV1 | 3.30E-05 | 13.925932 | Variant |
| rs34712979 | A | G | 0.254628 | -0.0287069 | 0.00198983 | 421986 | 3.50E-47 | FEV1 | 0.000187 | 78.925767 | Variant |
| rs35506 | A | T | 0.715534 | 0.0131164 | 0.00192867 | 421986 | 1.00E-11 | FEV1 | 4.50E-05 | 18.990135 | Variant |
| rs35945722 | A | G | 0.188457 | 0.0178664 | 0.00222614 | 421986 | 1.00E-15 | FEV1 | 4.70E-05 | 19.83418 | Variant |
| rs3790076 | T | G | 0.572955 | -0.0195938 | 0.00175665 | 421986 | 6.80E-29 | FEV1 | 0.000144 | 60.774448 | Variant |
| rs3791679 | G | A | 0.227266 | -0.0318929 | 0.00206608 | 421986 | 9.30E-54 | FEV1 | 0.000198 | 83.569379 | Variant |
| rs3814115 | C | T | 0.319922 | 0.0105084 | 0.00185925 | 421986 | 1.60E-08 | FEV1 | 3.30E-05 | 13.925932 | Variant |
| rs3846054 | T | C | 0.450793 | 0.00994881 | 0.00174162 | 421986 | 1.10E-08 | FEV1 | 3.80E-05 | 16.036001 | Variant |
| rs3858603 | A | G | 0.501593 | -0.0105008 | 0.00173339 | 421986 | 1.40E-09 | FEV1 | 4.30E-05 | 18.146092 | Variant |
| rs4079029 | T | C | 0.33955 | 0.0120784 | 0.00183566 | 421986 | 4.70E-11 | FEV1 | 4.60E-05 | 19.412157 | Variant |
| rs42032 | A | G | 0.262805 | 0.0144136 | 0.00197796 | 421986 | 3.20E-13 | FEV1 | 4.90E-05 | 20.678229 | Variant |
| rs425102 | G | T | 0.240797 | -0.0162187 | 0.00202529 | 421986 | 1.20E-15 | FEV1 | 5.60E-05 | 23.632427 | Variant |
| rs4320932 | C | T | 0.185678 | -0.0161835 | 0.00224453 | 421986 | 5.60E-13 | FEV1 | 3.70E-05 | 15.613986 | Variant |
| rs4413161 | A | T | 0.715755 | -0.0105207 | 0.00192117 | 421986 | 4.30E-08 | FEV1 | 2.90E-05 | 12.237891 | Variant |
| rs4489748 | C | T | 0.30689 | 0.011644 | 0.00188251 | 421986 | 6.20E-10 | FEV1 | 3.90E-05 | 16.458018 | Variant |
| rs4634390 | A | G | 0.278865 | 0.0117469 | 0.00193483 | 421986 | 1.30E-09 | FEV1 | 3.50E-05 | 14.769957 | Variant |
| rs4655027 | T | C | 0.575109 | -0.0122217 | 0.00175308 | 421986 | 3.10E-12 | FEV1 | 5.60E-05 | 23.632427 | Variant |
| rs4728358 | T | C | 0.687154 | -0.010465 | 0.00187807 | 421986 | 2.50E-08 | FEV1 | 3.20E-05 | 13.50392 | Variant |
| rs4785677 | G | A | 0.549173 | -0.010584 | 0.00174426 | 421986 | 1.30E-09 | FEV1 | 4.30E-05 | 18.146092 | Variant |
| rs4809328 | T | C | 0.667431 | -0.0128687 | 0.00184134 | 421986 | 2.80E-12 | FEV1 | 5.10E-05 | 21.522282 | Variant |
| rs4836528 | C | T | 0.409166 | -0.0153205 | 0.00178134 | 421986 | 7.90E-18 | FEV1 | 8.50E-05 | 35.871689 | Variant |
| rs4886778 | A | C | 0.494385 | 0.0155869 | 0.00173745 | 421986 | 2.90E-19 | FEV1 | 9.50E-05 | 40.092289 | Variant |
| rs4894559 | G | A | 0.733221 | -0.0117781 | 0.00196997 | 421986 | 2.20E-09 | FEV1 | 3.30E-05 | 13.925932 | Variant |
| rs4965600 | A | G | 0.612068 | -0.00975459 | 0.00178467 | 421986 | 4.60E-08 | FEV1 | 3.40E-05 | 14.347944 | Variant |
| rs513953 | G | A | 0.745133 | 0.0152258 | 0.00199806 | 421986 | 2.50E-14 | FEV1 | 5.20E-05 | 21.944309 | Variant |
| rs541601 | C | T | 0.81685 | 0.0137087 | 0.00224025 | 421986 | 9.40E-10 | FEV1 | 2.70E-05 | 11.393876 | Outlier |
| rs55775505 | T | C | 0.314794 | -0.0167461 | 0.00186897 | 421986 | 3.20E-19 | FEV1 | 8.20E-05 | 34.605526 | Variant |
| rs55831773 | T | C | 0.199579 | -0.0123999 | 0.00221625 | 421986 | 2.20E-08 | FEV1 | 2.40E-05 | 10.127859 | Variant |
| rs560805 | A | T | 0.67787 | -0.0103877 | 0.00185785 | 421986 | 2.30E-08 | FEV1 | 3.20E-05 | 13.50392 | Variant |
| rs56291455 | A | C | 0.384652 | -0.0115801 | 0.00178991 | 421986 | 9.80E-11 | FEV1 | 4.70E-05 | 19.83418 | Variant |
| rs56353224 | C | T | 0.371209 | -0.0104971 | 0.00183397 | 421986 | 1.00E-08 | FEV1 | 3.60E-05 | 15.191971 | Variant |
| rs56397594 | C | T | 0.21379 | 0.0129215 | 0.00211484 | 421986 | 1.00E-09 | FEV1 | 3.00E-05 | 12.6599 | Variant |
| rs56795193 | C | T | 0.202824 | 0.0136377 | 0.00219095 | 421986 | 4.80E-10 | FEV1 | 3.00E-05 | 12.6599 | Variant |
| rs5742643 | C | T | 0.752229 | -0.0134223 | 0.00199717 | 421986 | 1.80E-11 | FEV1 | 4.00E-05 | 16.880035 | Variant |
| rs591322 | G | T | 0.442937 | 0.0106529 | 0.00174897 | 421986 | 1.10E-09 | FEV1 | 4.30E-05 | 18.146092 | Variant |
| rs5992929 | T | C | 0.284928 | 0.0145607 | 0.00192981 | 421986 | 4.50E-14 | FEV1 | 5.50E-05 | 23.210397 | Outlier |
| rs60839038 | C | A | 0.47352 | 0.0110861 | 0.00176794 | 421986 | 3.60E-10 | FEV1 | 4.60E-05 | 19.412157 | Variant |
| rs6088619 | G | A | 0.135815 | 0.0182288 | 0.0025538 | 421986 | 9.50E-13 | FEV1 | 2.80E-05 | 11.815883 | Variant |
| rs6119786 | A | G | 0.156746 | -0.0148894 | 0.00239158 | 421986 | 4.80E-10 | FEV1 | 2.40E-05 | 10.127859 | Variant |
| rs6140000 | G | T | 0.497765 | -0.00990775 | 0.0017392 | 421986 | 1.20E-08 | FEV1 | 3.80E-05 | 16.036001 | Variant |
| rs61821293 | G | T | 0.218739 | 0.0125044 | 0.00209657 | 421986 | 2.50E-09 | FEV1 | 2.90E-05 | 12.237891 | Variant |
| rs62126408 | C | T | 0.143096 | 0.021851 | 0.00247954 | 421986 | 1.20E-18 | FEV1 | 4.50E-05 | 18.990135 | Variant |
| rs62621197 | T | C | 0.037085 | -0.0566593 | 0.00477416 | 421986 | 1.70E-32 | FEV1 | 2.40E-05 | 10.127859 | Variant |
| rs6429838 | G | A | 0.493347 | 0.0105519 | 0.00173342 | 421986 | 1.10E-09 | FEV1 | 4.40E-05 | 18.568113 | Variant |
| rs6473015 | C | A | 0.285558 | 0.0116677 | 0.00192246 | 421986 | 1.30E-09 | FEV1 | 3.60E-05 | 15.191971 | Variant |

| | | | | | | | | | | | |
|---|---|---|---|---|---|---|---|---|---|---|---|
| rs663378 | A | G | 0.833573 | -0.0159719 | 0.00235108 | 421986 | 1.10E-11 | FEV1 | 3.00E-05 | 12.6599 | Variant |
| rs6671370 | A | G | 0.287459 | 0.0183728 | 0.0019151 | 421986 | 8.50E-22 | FEV1 | 8.90E-05 | 37.559919 | Variant |
| rs6690055 | A | G | 0.253509 | -0.0166819 | 0.00198913 | 421986 | 5.00E-17 | FEV1 | 6.30E-05 | 26.586667 | Variant |
| rs6729308 | A | G | 0.438991 | -0.0150416 | 0.00175349 | 421986 | 9.60E-18 | FEV1 | 8.60E-05 | 36.293745 | Variant |
| rs67326273 | G | A | 0.192532 | 0.0133963 | 0.00220699 | 421986 | 1.30E-09 | FEV1 | 2.70E-05 | 11.393876 | Variant |
| rs6762578 | A | G | 0.77845 | 0.017359 | 0.00209804 | 421986 | 1.30E-16 | FEV1 | 5.60E-05 | 23.632427 | Outlier |
| rs6790568 | T | C | 0.174664 | -0.0147262 | 0.00228448 | 421986 | 1.10E-10 | FEV1 | 2.80E-05 | 11.815883 | Variant |
| rs681907 | C | T | 0.200558 | -0.0155049 | 0.00217293 | 421986 | 9.60E-13 | FEV1 | 3.90E-05 | 16.458018 | Variant |
| rs6902142 | G | A | 0.181862 | -0.0132249 | 0.00226845 | 421986 | 5.50E-09 | FEV1 | 2.40E-05 | 10.127859 | Variant |
| rs6923431 | C | A | 0.734557 | 0.013245 | 0.00194621 | 421986 | 1.00E-11 | FEV1 | 4.30E-05 | 18.146092 | Variant |
| rs6965423 | C | T | 0.50901 | -0.0102531 | 0.00173955 | 421986 | 3.80E-09 | FEV1 | 4.10E-05 | 17.302053 | Variant |
| rs7004355 | C | T | 0.463259 | -0.0158221 | 0.00174121 | 421986 | 1.00E-19 | FEV1 | 9.70E-05 | 40.936419 | Variant |
| rs7075773 | T | C | 0.410246 | -0.0128718 | 0.00176867 | 421986 | 3.40E-13 | FEV1 | 6.10E-05 | 25.742594 | Variant |
| rs7105777 | T | C | 0.631869 | -0.010458 | 0.0018051 | 421986 | 6.90E-09 | FEV1 | 3.70E-05 | 15.613986 | Variant |
| rs7124681 | A | C | 0.408392 | -0.0120528 | 0.00176185 | 421986 | 7.90E-12 | FEV1 | 5.40E-05 | 22.788367 | Variant |
| rs7132318 | C | T | 0.337297 | -0.0109793 | 0.00184173 | 421986 | 2.50E-09 | FEV1 | 3.80E-05 | 16.036001 | Variant |
| rs7211143 | G | A | 0.295757 | 0.0106612 | 0.00191077 | 421986 | 2.40E-08 | FEV1 | 3.10E-05 | 13.08191 | Variant |
| rs72643433 | A | G | 0.252489 | -0.0172939 | 0.00200221 | 421986 | 5.80E-18 | FEV1 | 6.70E-05 | 28.274822 | Variant |
| rs72666766 | G | A | 0.181385 | -0.0134569 | 0.00225303 | 421986 | 2.30E-09 | FEV1 | 2.50E-05 | 10.549864 | Variant |
| rs72718111 | C | A | 0.25821 | 0.0124661 | 0.00200909 | 421986 | 5.50E-10 | FEV1 | 3.50E-05 | 14.769957 | Variant |
| rs72755233 | A | G | 0.111547 | -0.0312731 | 0.00275452 | 421986 | 7.10E-30 | FEV1 | 6.10E-05 | 25.742594 | Variant |
| rs72904209 | C | T | 0.13427 | -0.0221176 | 0.00254798 | 421986 | 3.90E-18 | FEV1 | 4.20E-05 | 17.724072 | Variant |
| rs72976986 | A | G | 0.190197 | 0.0135612 | 0.00223359 | 421986 | 1.30E-09 | FEV1 | 2.70E-05 | 11.393876 | Variant |
| rs73154314 | C | A | 0.267598 | -0.0154831 | 0.00195515 | 421986 | 2.40E-15 | FEV1 | 5.80E-05 | 24.476492 | Variant |
| rs7336610 | T | C | 0.575324 | -0.011074 | 0.00176087 | 421986 | 3.20E-10 | FEV1 | 4.60E-05 | 19.412157 | Variant |
| rs735830 | A | G | 0.208865 | -0.0134849 | 0.0021364 | 421986 | 2.80E-10 | FEV1 | 3.10E-05 | 13.08191 | Variant |
| rs7359156 | G | A | 0.209384 | -0.013569 | 0.00214141 | 421986 | 2.40E-10 | FEV1 | 3.20E-05 | 13.50392 | Variant |
| rs7402983 | C | A | 0.601505 | -0.0113048 | 0.00179997 | 421986 | 3.40E-10 | FEV1 | 4.50E-05 | 18.990135 | Variant |
| rs7493429 | C | A | 0.304929 | 0.0124504 | 0.00189746 | 421986 | 5.30E-11 | FEV1 | 4.30E-05 | 18.146092 | Outlier |
| rs75216331 | A | G | 0.237224 | -0.0124016 | 0.00204074 | 421986 | 1.20E-09 | FEV1 | 3.20E-05 | 13.50392 | Variant |
| rs7558918 | G | T | 0.447756 | 0.0172521 | 0.00174429 | 421986 | 4.60E-23 | FEV1 | 0.000115 | 48.533741 | Variant |
| rs75831829 | T | C | 0.234053 | -0.0146225 | 0.0020589 | 421986 | 1.20E-12 | FEV1 | 4.30E-05 | 18.146092 | Variant |
| rs761422 | G | A | 0.49067 | 0.0118383 | 0.00173246 | 421986 | 8.30E-12 | FEV1 | 5.50E-05 | 23.210397 | Variant |
| rs7638143 | A | G | 0.582086 | -0.014996 | 0.0017596 | 421986 | 1.60E-17 | FEV1 | 8.40E-05 | 35.449634 | Outlier |
| rs7663740 | T | G | 0.542403 | 0.0285998 | 0.00173867 | 421986 | 8.50E-61 | FEV1 | 0.000318 | 134.233598 | Variant |
| rs7723049 | G | A | 0.198601 | -0.0149625 | 0.00218333 | 421986 | 7.20E-12 | FEV1 | 3.50E-05 | 14.769957 | Outlier |
| rs772920 | G | C | 0.335384 | -0.0123802 | 0.00183642 | 421986 | 1.60E-11 | FEV1 | 4.80E-05 | 20.256204 | Variant |
| rs7733410 | A | G | 0.442953 | 0.0202565 | 0.00174619 | 421986 | 4.10E-31 | FEV1 | 0.000157 | 66.261891 | Variant |
| rs777707 | A | G | 0.531166 | 0.012224 | 0.0017418 | 421986 | 2.30E-12 | FEV1 | 5.80E-05 | 24.476492 | Outlier |
| rs7778674 | G | A | 0.421388 | 0.013351 | 0.00175562 | 421986 | 2.90E-14 | FEV1 | 6.70E-05 | 28.274822 | Variant |
| rs77804065 | T | C | 0.224606 | -0.0296159 | 0.00209468 | 421986 | 2.20E-45 | FEV1 | 0.000165 | 69.63885 | Variant |
| rs78101726 | G | A | 0.152397 | -0.0225814 | 0.00241391 | 421986 | 8.40E-21 | FEV1 | 5.40E-05 | 22.788367 | Variant |
| rs784808 | C | T | 0.556276 | 0.00970754 | 0.00175016 | 421986 | 2.90E-08 | FEV1 | 3.60E-05 | 15.191971 | Variant |
| rs7854962 | G | C | 0.212959 | -0.0158986 | 0.00213883 | 421986 | 1.10E-13 | FEV1 | 4.40E-05 | 18.568113 | Variant |
| rs78579285 | T | C | 0.181123 | -0.0174186 | 0.00225239 | 421986 | 1.00E-14 | FEV1 | 4.20E-05 | 17.724072 | Variant |
| rs7930706 | A | G | 0.26492 | 0.0113551 | 0.00197209 | 421986 | 8.50E-09 | FEV1 | 3.10E-05 | 13.08191 | Variant |
| rs7961994 | T | A | 0.609755 | 0.0112438 | 0.00177642 | 421986 | 2.50E-10 | FEV1 | 4.50E-05 | 18.990135 | Variant |

| | | | | | | | | | | | | |
|---|---|---|---|---|---|---|---|---|---|---|---|---|
| rs7968682 | T | G | 0.517742 | -0.0239081 | 0.00174147 | 421986 | 6.80E-43 | FEV1 | 0.000223 | 94.123422 | Variant |
| rs8034236 | T | C | 0.624791 | 0.00983122 | 0.00179312 | 421986 | 4.20E-08 | FEV1 | 3.30E-05 | 13.925932 | Variant |
| rs803765 | A | C | 0.346274 | -0.0115526 | 0.00182565 | 421986 | 2.50E-10 | FEV1 | 4.30E-05 | 18.146092 | Outlier |
| rs8062719 | G | A | 0.635212 | 0.0128509 | 0.00181379 | 421986 | 1.40E-12 | FEV1 | 5.50E-05 | 23.210397 | Variant |
| rs8069671 | T | G | 0.390081 | -0.0125899 | 0.00177871 | 421986 | 1.50E-12 | FEV1 | 5.60E-05 | 23.632427 | Variant |
| rs841217 | C | T | 0.542519 | -0.0102795 | 0.00174789 | 421986 | 4.10E-09 | FEV1 | 4.10E-05 | 17.302053 | Variant |
| rs853678 | A | T | 0.163236 | -0.0214029 | 0.00234368 | 421986 | 6.70E-20 | FEV1 | 5.40E-05 | 22.788367 | Variant |
| rs876531 | T | C | 0.455993 | -0.016464 | 0.00174605 | 421986 | 4.10E-21 | FEV1 | 0.000105 | 44.312973 | Variant |
| rs879098 | G | A | 0.39884 | -0.0113983 | 0.00177623 | 421986 | 1.40E-10 | FEV1 | 4.70E-05 | 19.83418 | Variant |
| rs879394 | T | G | 0.233436 | -0.0190885 | 0.00205722 | 421986 | 1.70E-20 | FEV1 | 7.30E-05 | 30.807081 | Variant |
| rs889312 | A | C | 0.715882 | 0.010585 | 0.00192498 | 421986 | 3.80E-08 | FEV1 | 2.90E-05 | 12.237891 | Variant |
| rs9295 | A | G | 0.295687 | 0.0117931 | 0.00190411 | 421986 | 5.90E-10 | FEV1 | 3.80E-05 | 16.036001 | Variant |
| rs9310389 | T | C | 0.5454 | 0.0100077 | 0.00174847 | 421986 | 1.00E-08 | FEV1 | 3.80E-05 | 16.036001 | Variant |
| rs9322332 | A | C | 0.45633 | 0.013407 | 0.00174017 | 421986 | 1.30E-14 | FEV1 | 7.00E-05 | 29.540948 | Variant |
| rs9864975 | G | A | 0.24399 | 0.0167741 | 0.00202079 | 421986 | 1.00E-16 | FEV1 | 6.00E-05 | 25.320559 | Variant |
| rs9886260 | A | G | 0.722783 | -0.0128429 | 0.00194321 | 421986 | 3.90E-11 | FEV1 | 4.10E-05 | 17.302053 | Variant |
| rs9912553 | G | C | 0.723438 | 0.0199953 | 0.00194048 | 421986 | 6.70E-25 | FEV1 | 0.000101 | 42.624689 | Variant |
| rs9988963 | G | C | 0.737251 | 0.0109336 | 0.00197337 | 421986 | 3.00E-08 | FEV1 | 2.80E-05 | 11.815883 | Variant |

FEV1, forced expiratory volume in one second; SNP, single nucleotide polymorphism; IVW, inverse-variance weighted.

**Table S3.** Genetic instruments for FVC.

| SNP | Effect allele | Other allele | Effect allele frequency | Beta | Standard error | Sample size | P value | Exposure | R2 | F statistic | Outliers (Radial IVW) |
|---|---|---|---|---|---|---|---|---|---|---|---|
| rs10141786 | G | A | 0.596493 | -0.0135681 | 0.00168175 | 421986 | 7.20E-16 | FVC | 7.40E-05 | 31.229127 | Variant |
| rs10202701 | T | C | 0.541923 | 0.0115889 | 0.00165011 | 421986 | 2.20E-12 | FVC | 5.80E-05 | 24.476492 | Variant |
| rs1054661 | C | T | 0.342598 | 0.0101651 | 0.00172786 | 421986 | 4.00E-09 | FVC | 3.70E-05 | 15.613986 | Variant |
| rs10761495 | C | A | 0.597215 | 0.0117777 | 0.00167988 | 421986 | 2.40E-12 | FVC | 5.60E-05 | 23.632427 | Variant |
| rs10789246 | C | T | 0.301089 | -0.0123427 | 0.00178506 | 421986 | 4.70E-12 | FVC | 4.80E-05 | 20.256204 | Variant |
| rs10797116 | C | T | 0.542079 | -0.0121521 | 0.00165228 | 421986 | 1.90E-13 | FVC | 6.40E-05 | 27.008705 | Variant |
| rs10853628 | C | T | 0.384831 | -0.0134021 | 0.00168952 | 421986 | 2.10E-15 | FVC | 7.10E-05 | 29.962991 | Variant |
| rs10861686 | T | C | 0.571827 | -0.00930194 | 0.00166073 | 421986 | 2.10E-08 | FVC | 3.60E-05 | 15.191971 | Variant |
| rs10874340 | T | G | 0.493361 | 0.0103061 | 0.00163772 | 421986 | 3.10E-10 | FVC | 4.70E-05 | 19.83418 | Variant |
| rs10892061 | T | C | 0.655603 | 0.010252 | 0.00172686 | 421986 | 2.90E-09 | FVC | 3.80E-05 | 16.036001 | Variant |
| rs10916137 | A | G | 0.170792 | -0.0161307 | 0.00218459 | 421986 | 1.50E-13 | FVC | 3.70E-05 | 15.613986 | Variant |
| rs11000765 | C | G | 0.720336 | -0.0112364 | 0.00182542 | 421986 | 7.50E-10 | FVC | 3.60E-05 | 15.191971 | Outlier |
| rs111365325 | T | C | 0.231244 | -0.0185706 | 0.00194997 | 421986 | 1.70E-21 | FVC | 7.60E-05 | 32.073222 | Variant |
| rs11164653 | C | T | 0.59054 | -0.0114085 | 0.00167402 | 421986 | 9.40E-12 | FVC | 5.30E-05 | 22.366337 | Variant |
| rs11191818 | G | T | 0.515639 | 0.0114454 | 0.00165336 | 421986 | 4.40E-12 | FVC | 5.70E-05 | 24.054459 | Variant |
| rs11259936 | C | A | 0.52286 | 0.0173043 | 0.00164414 | 421986 | 6.60E-26 | FVC | 0.000131 | 55.287147 | Variant |
| rs11621587 | C | G | 0.183978 | 0.0212612 | 0.00211823 | 421986 | 1.00E-23 | FVC | 7.20E-05 | 30.385036 | Outlier |
| rs11632611 | A | G | 0.658418 | -0.011446 | 0.00175909 | 421986 | 7.70E-11 | FVC | 4.50E-05 | 18.990135 | Variant |
| rs11637681 | G | A | 0.276249 | -0.0114817 | 0.00185184 | 421986 | 5.60E-10 | FVC | 3.60E-05 | 15.191971 | Variant |
| rs11645649 | G | C | 0.593954 | 0.0107058 | 0.00170463 | 421986 | 3.40E-10 | FVC | 4.50E-05 | 18.990135 | Variant |
| rs11664552 | C | T | 0.300477 | 0.0103612 | 0.00180111 | 421986 | 8.80E-09 | FVC | 3.30E-05 | 13.925932 | Variant |
| rs11668582 | T | C | 0.390878 | 0.0138094 | 0.00168764 | 421986 | 2.80E-16 | FVC | 7.60E-05 | 32.073222 | Variant |
| rs11698277 | T | C | 0.319648 | 0.016012 | 0.00176946 | 421986 | 1.40E-19 | FVC | 8.40E-05 | 35.449634 | Outlier |
| rs11867847 | G | A | 0.505622 | 0.00976717 | 0.00164721 | 421986 | 3.00E-09 | FVC | 4.20E-05 | 17.724072 | Variant |
| rs11877758 | G | T | 0.3121 | -0.010769 | 0.00178155 | 421986 | 1.50E-09 | FVC | 3.70E-05 | 15.613986 | Outlier |
| rs11896404 | T | C | 0.38932 | 0.0097226 | 0.00168555 | 421986 | 8.00E-09 | FVC | 3.70E-05 | 15.613986 | Variant |
| rs11909473 | G | A | 0.28953 | 0.0107787 | 0.00182218 | 421986 | 3.30E-09 | FVC | 3.40E-05 | 14.347944 | Variant |
| rs11938781 | C | T | 0.165403 | -0.036626 | 0.00221596 | 421986 | 2.30E-61 | FVC | 0.000179 | 75.548659 | Variant |
| rs11967850 | T | C | 0.203699 | -0.0117444 | 0.00203397 | 421986 | 7.70E-09 | FVC | 2.60E-05 | 10.971869 | Variant |
| rs12051245 | C | T | 0.231666 | 0.0125612 | 0.00194877 | 421986 | 1.20E-10 | FVC | 3.50E-05 | 14.769957 | Variant |
| rs12052537 | A | C | 0.20591 | -0.0145821 | 0.00203664 | 421986 | 8.10E-13 | FVC | 4.00E-05 | 16.880035 | Variant |
| rs12054747 | T | C | 0.216455 | 0.0112373 | 0.0019943 | 421986 | 1.80E-08 | FVC | 2.60E-05 | 10.971869 | Variant |
| rs12099669 | A | G | 0.696156 | 0.0127696 | 0.00178372 | 421986 | 8.10E-13 | FVC | 5.10E-05 | 21.522282 | Variant |
| rs12139563 | G | A | 0.5652 | -0.014803 | 0.00165293 | 421986 | 3.40E-19 | FVC | 9.30E-05 | 39.248162 | Variant |
| rs12152266 | T | C | 0.27447 | 0.0143563 | 0.00183934 | 421986 | 5.90E-15 | FVC | 5.80E-05 | 24.476492 | Variant |
| rs12348523 | A | G | 0.156387 | 0.0148259 | 0.00226513 | 421986 | 5.90E-11 | FVC | 2.70E-05 | 11.393876 | Variant |
| rs12358810 | A | G | 0.392273 | -0.0131936 | 0.00168993 | 421986 | 5.80E-15 | FVC | 6.90E-05 | 29.118905 | Variant |
| rs12402966 | A | G | 0.213339 | 0.0202071 | 0.00199879 | 421986 | 5.00E-24 | FVC | 8.10E-05 | 34.183473 | Variant |
| rs12430764 | A | G | 0.512974 | -0.0100933 | 0.00164288 | 421986 | 8.10E-10 | FVC | 4.50E-05 | 18.990135 | Variant |
| rs12452590 | G | T | 0.361692 | 0.0110851 | 0.00172892 | 421986 | 1.40E-10 | FVC | 4.50E-05 | 18.990135 | Variant |
| rs12486605 | C | T | 0.428162 | 0.00910894 | 0.00165849 | 421986 | 4.00E-08 | FVC | 3.50E-05 | 14.769957 | Variant |
| rs1250535 | G | C | 0.675489 | -0.0117947 | 0.00176235 | 421986 | 2.20E-11 | FVC | 4.70E-05 | 19.83418 | Variant |

| | | | | | | | | | | | | |
|---|---|---|---|---|---|---|---|---|---|---|---|---|
| rs12571363 | A | C | 0.10664 | -0.0297543 | 0.00266251 | 421986 | 5.40E-29 | FVC | 5.60E-05 | 23.632427 | Variant |
| rs12614240 | T | C | 0.433883 | -0.0156274 | 0.00165606 | 421986 | 3.90E-21 | FVC | 0.000104 | 43.890901 | Variant |
| rs12641030 | G | A | 0.467529 | -0.0110493 | 0.00164454 | 421986 | 1.80E-11 | FVC | 5.30E-05 | 22.366337 | Variant |
| rs12684650 | T | C | 0.316579 | -0.0207794 | 0.00176534 | 421986 | 5.50E-32 | FVC | 0.000142 | 59.930238 | Variant |
| rs12713685 | C | T | 0.374424 | -0.0110318 | 0.00169411 | 421986 | 7.40E-11 | FVC | 4.70E-05 | 19.83418 | Variant |
| rs12790261 | A | C | 0.082405 | -0.0447471 | 0.00299096 | 421986 | 1.30E-50 | FVC | 8.00E-05 | 33.761421 | Variant |
| rs12985660 | C | T | 0.475562 | -0.0103226 | 0.00165161 | 421986 | 4.10E-10 | FVC | 4.60E-05 | 19.412157 | Variant |
| rs1299113 | A | G | 0.5241 | 0.0102322 | 0.00165011 | 421986 | 5.60E-10 | FVC | 4.50E-05 | 18.990135 | Variant |
| rs13023718 | G | A | 0.66664 | 0.0100463 | 0.00174447 | 421986 | 8.50E-09 | FVC | 3.50E-05 | 14.769957 | Variant |
| rs13108218 | G | A | 0.614862 | 0.0148225 | 0.00169865 | 421986 | 2.60E-18 | FVC | 8.50E-05 | 35.871689 | Outlier |
| rs13156484 | A | G | 0.472145 | -0.0142036 | 0.00166004 | 421986 | 1.20E-17 | FVC | 8.60E-05 | 36.293745 | Outlier |
| rs13229167 | A | G | 0.271146 | -0.0205426 | 0.00185147 | 421986 | 1.30E-28 | FVC | 0.000115 | 48.533741 | Variant |
| rs1344555 | T | C | 0.20277 | -0.0184191 | 0.00203961 | 421986 | 1.70E-19 | FVC | 6.20E-05 | 26.16463 | Variant |
| rs13723 | G | A | 0.491918 | -0.0100373 | 0.00164308 | 421986 | 1.00E-09 | FVC | 4.40E-05 | 18.568113 | Variant |
| rs1415701 | A | G | 0.267657 | -0.0230451 | 0.00187521 | 421986 | 1.00E-34 | FVC | 0.00014 | 59.086032 | Variant |
| rs1474419 | C | T | 0.577669 | -0.00937242 | 0.00166461 | 421986 | 1.80E-08 | FVC | 3.70E-05 | 15.613986 | Variant |
| rs1474647 | T | C | 0.550866 | -0.0134877 | 0.0016468 | 421986 | 2.60E-16 | FVC | 7.90E-05 | 33.33937 | Variant |
| rs1490384 | T | C | 0.500495 | 0.0246328 | 0.00163964 | 421986 | 5.20E-51 | FVC | 0.000267 | 112.69982 | Variant |
| rs149542 | A | G | 0.375642 | -0.0114549 | 0.00169827 | 421986 | 1.50E-11 | FVC | 5.10E-05 | 21.522282 | Variant |
| rs1516890 | A | G | 0.469749 | -0.0114366 | 0.00165471 | 421986 | 4.80E-12 | FVC | 5.60E-05 | 23.632427 | Variant |
| rs1555211 | T | C | 0.292035 | 0.014396 | 0.00180824 | 421986 | 1.70E-15 | FVC | 6.20E-05 | 26.16463 | Outlier |
| rs157595 | G | A | 0.616263 | 0.0116135 | 0.00173044 | 421986 | 1.90E-11 | FVC | 5.00E-05 | 21.100255 | Variant |
| rs1608592 | A | G | 0.261845 | -0.018905 | 0.00187413 | 421986 | 6.30E-24 | FVC | 9.30E-05 | 39.248162 | Variant |
| rs1637746 | A | G | 0.226569 | -0.0110712 | 0.00196778 | 421986 | 1.80E-08 | FVC | 2.60E-05 | 10.971869 | Variant |
| rs1656373 | T | C | 0.55742 | -0.0172006 | 0.00165265 | 421986 | 2.30E-25 | FVC | 0.000127 | 53.598775 | Variant |
| rs1668091 | C | T | 0.31711 | 0.0124279 | 0.00177541 | 421986 | 2.60E-12 | FVC | 5.00E-05 | 21.100255 | Variant |
| rs17387279 | G | T | 0.181103 | -0.0143769 | 0.0021417 | 421986 | 1.90E-11 | FVC | 3.20E-05 | 13.50392 | Variant |
| rs1818116 | G | A | 0.381268 | -0.00974805 | 0.00170614 | 421986 | 1.10E-08 | FVC | 3.60E-05 | 15.191971 | Variant |
| rs1966265 | A | G | 0.245129 | 0.0152999 | 0.00190502 | 421986 | 9.60E-16 | FVC | 5.70E-05 | 24.054459 | Variant |
| rs1983753 | A | G | 0.851273 | 0.0155112 | 0.00231937 | 421986 | 2.30E-11 | FVC | 2.70E-05 | 11.393876 | Variant |
| rs1984246 | A | C | 0.42304 | 0.0128671 | 0.00166162 | 421986 | 9.70E-15 | FVC | 6.90E-05 | 29.118905 | Variant |
| rs2005172 | C | A | 0.64023 | 0.0219024 | 0.0017318 | 421986 | 1.20E-36 | FVC | 0.000175 | 73.860126 | Outlier |
| rs2007530 | T | C | 0.173998 | 0.0202027 | 0.00217301 | 421986 | 1.40E-20 | FVC | 5.90E-05 | 24.898525 | Variant |
| rs2118022 | C | T | 0.360318 | -0.0114524 | 0.00171628 | 421986 | 2.50E-11 | FVC | 4.90E-05 | 20.678229 | Variant |
| rs2131354 | A | G | 0.526265 | 0.0230512 | 0.00164614 | 421986 | 1.50E-44 | FVC | 0.000232 | 97.923006 | Variant |
| rs2165241 | C | T | 0.508667 | 0.0196955 | 0.00164442 | 421986 | 4.70E-33 | FVC | 0.00017 | 71.749477 | Variant |
| rs2168704 | A | G | 0.561551 | 0.0106636 | 0.001652 | 421986 | 1.10E-10 | FVC | 4.90E-05 | 20.678229 | Variant |
| rs2181246 | A | T | 0.315032 | -0.0105367 | 0.00176834 | 421986 | 2.50E-09 | FVC | 3.60E-05 | 15.191971 | Variant |
| rs2186288 | A | G | 0.369909 | -0.0111102 | 0.00170446 | 421986 | 7.10E-11 | FVC | 4.70E-05 | 19.83418 | Outlier |
| rs2194411 | A | G | 0.128337 | 0.0278083 | 0.00248353 | 421986 | 4.20E-29 | FVC | 6.60E-05 | 27.852782 | Variant |
| rs2219320 | C | T | 0.253726 | -0.015474 | 0.00188065 | 421986 | 1.90E-16 | FVC | 6.10E-05 | 25.742594 | Variant |
| rs2273101 | T | C | 0.245563 | 0.021089 | 0.00190985 | 421986 | 2.40E-28 | FVC | 0.000107 | 45.15712 | Variant |
| rs2315007 | C | T | 0.667421 | -0.0146598 | 0.00174235 | 421986 | 4.00E-17 | FVC | 7.40E-05 | 31.229127 | Variant |
| rs2355595 | T | C | 0.353472 | 0.0096053 | 0.00171679 | 421986 | 2.20E-08 | FVC | 3.40E-05 | 14.347944 | Variant |
| rs2433733 | A | G | 0.677805 | 0.0100332 | 0.00175482 | 421986 | 1.10E-08 | FVC | 3.40E-05 | 14.347944 | Variant |
| rs246185 | C | T | 0.32244 | 0.0125927 | 0.001774 | 421986 | 1.30E-12 | FVC | 5.20E-05 | 21.944309 | Variant |
| rs2468964 | G | T | 0.60054 | 0.00965778 | 0.00167483 | 421986 | 8.10E-09 | FVC | 3.80E-05 | 16.036001 | Outlier |

| | | | | | | | | | | | | |
|---|---|---|---|---|---|---|---|---|---|---|---|---|
| rs2602691 | G | A | 0.449662 | -0.0108873 | 0.00166378 | 421986 | 6.00E-11 | FVC | 5.00E-05 | 21.100255 | Variant |
| rs2645977 | A | G | 0.357445 | 0.00992959 | 0.00171149 | 421986 | 6.60E-09 | FVC | 3.70E-05 | 15.613986 | Variant |
| rs2648725 | A | T | 0.213148 | 0.0171275 | 0.00200708 | 421986 | 1.40E-17 | FVC | 5.80E-05 | 24.476492 | Variant |
| rs2679053 | G | A | 0.599214 | 0.00937567 | 0.00167921 | 421986 | 2.40E-08 | FVC | 3.50E-05 | 14.769957 | Variant |
| rs2717329 | A | G | 0.294673 | 0.014239 | 0.0018328 | 421986 | 7.90E-15 | FVC | 5.90E-05 | 24.898525 | Variant |
| rs2760402 | C | A | 0.649252 | 0.00977621 | 0.00172753 | 421986 | 1.50E-08 | FVC | 3.50E-05 | 14.769957 | Variant |
| rs2780226 | T | C | 0.910509 | -0.0426163 | 0.0028699 | 421986 | 7.00E-50 | FVC | 8.50E-05 | 35.871689 | Variant |
| rs2807339 | C | T | 0.758119 | 0.0126736 | 0.00191263 | 421986 | 3.40E-11 | FVC | 3.80E-05 | 16.036001 | Variant |
| rs2808096 | G | A | 0.21446 | 0.0110218 | 0.0020005 | 421986 | 3.60E-08 | FVC | 2.40E-05 | 10.127859 | Variant |
| rs2821334 | T | G | 0.44435 | 0.0096939 | 0.00164775 | 421986 | 4.00E-09 | FVC | 4.10E-05 | 17.302053 | Variant |
| rs28391281 | C | T | 0.456626 | -0.0108229 | 0.00164482 | 421986 | 4.70E-11 | FVC | 5.10E-05 | 21.522282 | Variant |
| rs28409044 | A | C | 0.333596 | 0.0128599 | 0.00174168 | 421986 | 1.50E-13 | FVC | 5.70E-05 | 24.054459 | Variant |
| rs28427480 | C | A | 0.093765 | 0.0395816 | 0.00282649 | 421986 | 1.50E-44 | FVC | 7.90E-05 | 33.33937 | Variant |
| rs28444681 | A | C | 0.255224 | 0.0114328 | 0.00188044 | 421986 | 1.20E-09 | FVC | 3.30E-05 | 13.925932 | Variant |
| rs2871960 | C | A | 0.444976 | 0.0197056 | 0.00165006 | 421986 | 7.10E-33 | FVC | 0.000167 | 70.483099 | Variant |
| rs2882485 | G | A | 0.527663 | -0.0115807 | 0.00164247 | 421986 | 1.80E-12 | FVC | 5.90E-05 | 24.898525 | Variant |
| rs2885697 | T | G | 0.665178 | -0.0207198 | 0.00173474 | 421986 | 7.00E-33 | FVC | 0.000151 | 63.729207 | Variant |
| rs309137 | C | T | 0.227462 | 0.0154018 | 0.00194955 | 421986 | 2.80E-15 | FVC | 5.20E-05 | 21.944309 | Variant |
| rs3116605 | C | T | 0.215238 | -0.0153157 | 0.00199894 | 421986 | 1.80E-14 | FVC | 4.70E-05 | 19.83418 | Variant |
| rs3253 | T | C | 0.314156 | 0.0133966 | 0.00176807 | 421986 | 3.50E-14 | FVC | 5.90E-05 | 24.898525 | Variant |
| rs344133 | A | G | 0.614205 | 0.0111994 | 0.00168384 | 421986 | 2.90E-11 | FVC | 5.00E-05 | 21.100255 | Outlier |
| rs34587839 | A | G | 0.154712 | -0.0191516 | 0.00227501 | 421986 | 3.80E-17 | FVC | 4.40E-05 | 18.568113 | Outlier |
| rs34810766 | T | C | 0.188747 | 0.0123596 | 0.00210106 | 421986 | 4.00E-09 | FVC | 2.50E-05 | 10.549864 | Variant |
| rs35344256 | A | C | 0.305163 | -0.0173682 | 0.0017847 | 421986 | 2.20E-22 | FVC | 9.50E-05 | 40.092289 | Variant |
| rs35344761 | A | C | 0.122131 | -0.0192934 | 0.00252637 | 421986 | 2.20E-14 | FVC | 3.00E-05 | 12.6599 | Variant |
| rs35480566 | G | A | 0.439723 | 0.0149967 | 0.0016583 | 421986 | 1.50E-19 | FVC | 9.60E-05 | 40.514353 | Outlier |
| rs35506 | A | T | 0.715534 | 0.0135949 | 0.00182407 | 421986 | 9.10E-14 | FVC | 5.40E-05 | 22.788367 | Variant |
| rs35668185 | C | T | 0.207881 | -0.0115562 | 0.00201805 | 421986 | 1.00E-08 | FVC | 2.60E-05 | 10.971869 | Variant |
| rs35805829 | C | T | 0.346839 | -0.0133102 | 0.00172266 | 421986 | 1.10E-14 | FVC | 6.40E-05 | 27.008705 | Variant |
| rs3732987 | A | C | 0.524508 | 0.0105337 | 0.00164918 | 421986 | 1.70E-10 | FVC | 4.80E-05 | 20.256204 | Variant |
| rs3734254 | T | C | 0.790532 | 0.0207412 | 0.00201522 | 421986 | 7.60E-25 | FVC | 8.30E-05 | 35.027579 | Variant |
| rs3743347 | A | C | 0.231535 | -0.0169457 | 0.00194802 | 421986 | 3.40E-18 | FVC | 6.40E-05 | 27.008705 | Variant |
| rs3790076 | T | G | 0.572955 | -0.0208074 | 0.00166216 | 421986 | 5.90E-36 | FVC | 0.000182 | 76.815068 | Variant |
| rs3791679 | G | A | 0.227266 | -0.0417426 | 0.00195359 | 421986 | 2.70E-101 | FVC | 0.00038 | 160.41488 | Variant |
| rs3806443 | G | C | 0.265329 | 0.0108763 | 0.00186138 | 421986 | 5.10E-09 | FVC | 3.20E-05 | 13.50392 | Variant |
| rs3814316 | A | G | 0.368453 | -0.00946144 | 0.00170087 | 421986 | 2.70E-08 | FVC | 3.40E-05 | 14.347944 | Variant |
| rs3828559 | G | A | 0.758433 | 0.0117356 | 0.00191612 | 421986 | 9.10E-10 | FVC | 3.30E-05 | 13.925932 | Variant |
| rs3858603 | A | G | 0.501593 | -0.012366 | 0.00163937 | 421986 | 4.60E-14 | FVC | 6.70E-05 | 28.274822 | Variant |
| rs42038 | T | C | 0.298973 | 0.0162055 | 0.001795 | 421986 | 1.70E-19 | FVC | 8.10E-05 | 34.183473 | Variant |
| rs4244808 | G | T | 0.459999 | 0.0116467 | 0.00165946 | 421986 | 2.20E-12 | FVC | 5.80E-05 | 24.476492 | Variant |
| rs425102 | G | T | 0.240797 | -0.0194207 | 0.00191587 | 421986 | 3.80E-24 | FVC | 8.90E-05 | 37.559919 | Variant |
| rs4497878 | C | T | 0.715464 | -0.0146752 | 0.00181627 | 421986 | 6.50E-16 | FVC | 6.30E-05 | 26.586667 | Variant |
| rs4650619 | T | C | 0.658131 | 0.0110735 | 0.00173535 | 421986 | 1.80E-10 | FVC | 4.30E-05 | 18.146092 | Variant |
| rs4693974 | G | A | 0.574007 | 0.0115384 | 0.00166303 | 421986 | 4.00E-12 | FVC | 5.60E-05 | 23.632427 | Variant |
| rs4700485 | G | A | 0.719912 | 0.0108331 | 0.00182997 | 421986 | 3.20E-09 | FVC | 3.30E-05 | 13.925932 | Variant |
| rs4700662 | T | C | 0.664035 | 0.0130245 | 0.00174731 | 421986 | 9.10E-14 | FVC | 5.90E-05 | 24.898525 | Variant |
| rs4713135 | A | G | 0.237248 | -0.0160487 | 0.00192704 | 421986 | 8.20E-17 | FVC | 5.90E-05 | 24.898525 | Outlier |

| | | | | | | | | | | | | |
|---|---|---|---|---|---|---|---|---|---|---|---|---|
| rs4728358 | T | C | 0.687154 | -0.0105408 | 0.0017764 | 421986 | 3.00E-09 | FVC | 3.60E-05 | 15.191971 | Variant |
| rs4735766 | T | G | 0.285258 | 0.0149222 | 0.00181989 | 421986 | 2.40E-16 | FVC | 6.50E-05 | 27.430743 | Variant |
| rs4759316 | A | G | 0.57091 | 0.0104193 | 0.00165644 | 421986 | 3.20E-10 | FVC | 4.60E-05 | 19.412157 | Variant |
| rs4785677 | G | A | 0.549173 | -0.00984707 | 0.00165043 | 421986 | 2.40E-09 | FVC | 4.20E-05 | 17.724072 | Variant |
| rs4794006 | A | G | 0.555023 | -0.010443 | 0.00165309 | 421986 | 2.70E-10 | FVC | 4.70E-05 | 19.83418 | Variant |
| rs4909017 | T | C | 0.557895 | 0.0127025 | 0.00165126 | 421986 | 1.40E-14 | FVC | 6.90E-05 | 29.118905 | Variant |
| rs4924413 | A | G | 0.283479 | 0.0108259 | 0.00182023 | 421986 | 2.70E-09 | FVC | 3.40E-05 | 14.347944 | Variant |
| rs4951407 | G | T | 0.661152 | 0.0115513 | 0.00173213 | 421986 | 2.60E-11 | FVC | 4.70E-05 | 19.83418 | Variant |
| rs55681913 | C | T | 0.10621 | 0.0206406 | 0.00269286 | 421986 | 1.80E-14 | FVC | 2.60E-05 | 10.971869 | Variant |
| rs55758152 | A | G | 0.325764 | 0.0108471 | 0.00177131 | 421986 | 9.10E-10 | FVC | 3.90E-05 | 16.458018 | Variant |
| rs55775505 | T | C | 0.314794 | -0.014816 | 0.00176743 | 421986 | 5.20E-17 | FVC | 7.20E-05 | 30.385036 | Variant |
| rs55778236 | C | T | 0.27895 | 0.013512 | 0.00183002 | 421986 | 1.50E-13 | FVC | 5.20E-05 | 21.944309 | Variant |
| rs558107 | G | A | 0.587806 | -0.0109259 | 0.00167201 | 421986 | 6.40E-11 | FVC | 4.90E-05 | 20.678229 | Variant |
| rs55831773 | T | C | 0.199579 | -0.0143551 | 0.00209681 | 421986 | 7.60E-12 | FVC | 3.50E-05 | 14.769957 | Variant |
| rs56099233 | T | G | 0.203137 | 0.0119603 | 0.00205819 | 421986 | 6.20E-09 | FVC | 2.60E-05 | 10.971869 | Variant |
| rs57072412 | A | G | 0.383191 | 0.010773 | 0.00168954 | 421986 | 1.80E-10 | FVC | 4.60E-05 | 19.412157 | Variant |
| rs5742643 | C | T | 0.752229 | -0.0147931 | 0.00188885 | 421986 | 4.80E-15 | FVC | 5.40E-05 | 22.788367 | Variant |
| rs575837 | G | A | 0.784728 | 0.0127629 | 0.0020075 | 421986 | 2.00E-10 | FVC | 3.20E-05 | 13.50392 | Outlier |
| rs582780 | G | A | 0.422609 | 0.0125544 | 0.0016756 | 421986 | 6.80E-14 | FVC | 6.50E-05 | 27.430743 | Variant |
| rs59032271 | T | C | 0.467565 | -0.0102701 | 0.00164133 | 421986 | 3.90E-10 | FVC | 4.60E-05 | 19.412157 | Variant |
| rs5992929 | T | C | 0.284928 | 0.0107963 | 0.0018267 | 421986 | 3.40E-09 | FVC | 3.40E-05 | 14.347944 | Outlier |
| rs60839038 | C | A | 0.47352 | 0.0125201 | 0.00167309 | 421986 | 7.30E-14 | FVC | 6.60E-05 | 27.852782 | Variant |
| rs6088619 | G | A | 0.135815 | 0.0175247 | 0.00241661 | 421986 | 4.10E-13 | FVC | 2.90E-05 | 12.237891 | Variant |
| rs6140000 | G | T | 0.497765 | -0.00979911 | 0.00164577 | 421986 | 2.60E-09 | FVC | 4.20E-05 | 17.724072 | Variant |
| rs62515437 | T | G | 0.225213 | 0.0156907 | 0.00196681 | 421986 | 1.50E-15 | FVC | 5.30E-05 | 22.366337 | Variant |
| rs62621197 | T | C | 0.037085 | -0.0676827 | 0.00451793 | 421986 | 9.80E-51 | FVC | 3.80E-05 | 16.036001 | Variant |
| rs644570 | A | G | 0.632228 | 0.0105147 | 0.00170914 | 421986 | 7.60E-10 | FVC | 4.20E-05 | 17.724072 | Variant |
| rs6487686 | C | T | 0.772904 | -0.0171409 | 0.00196297 | 421986 | 2.50E-18 | FVC | 6.30E-05 | 26.586667 | Variant |
| rs6501431 | T | C | 0.784491 | 0.01294 | 0.00199944 | 421986 | 9.70E-11 | FVC | 3.40E-05 | 14.347944 | Variant |
| rs661857 | C | T | 0.482472 | 0.0148279 | 0.00164183 | 421986 | 1.70E-19 | FVC | 9.70E-05 | 40.936419 | Variant |
| rs663378 | A | G | 0.833573 | -0.0151571 | 0.00222469 | 421986 | 9.50E-12 | FVC | 3.10E-05 | 13.08191 | Variant |
| rs6658835 | G | A | 0.269388 | 0.019046 | 0.00185345 | 421986 | 9.00E-25 | FVC | 9.90E-05 | 41.780552 | Variant |
| rs6671370 | A | G | 0.287459 | 0.0174763 | 0.00181023 | 421986 | 4.70E-22 | FVC | 9.00E-05 | 37.981978 | Variant |
| rs668844 | A | C | 0.482052 | 0.00944665 | 0.00164055 | 421986 | 8.50E-09 | FVC | 3.90E-05 | 16.458018 | Variant |
| rs67232546 | T | C | 0.21257 | -0.0120519 | 0.0020216 | 421986 | 2.50E-09 | FVC | 2.80E-05 | 11.815883 | Variant |
| rs6762578 | A | G | 0.77845 | 0.0195725 | 0.00198462 | 421986 | 6.10E-23 | FVC | 8.00E-05 | 33.761421 | Outlier |
| rs6803045 | A | G | 0.582831 | 0.0104255 | 0.00167546 | 421986 | 4.90E-10 | FVC | 4.50E-05 | 18.990135 | Variant |
| rs6849555 | C | G | 0.201305 | 0.0136053 | 0.00210903 | 421986 | 1.10E-10 | FVC | 3.20E-05 | 13.50392 | Variant |
| rs6886191 | G | A | 0.559405 | 0.0111534 | 0.00165843 | 421986 | 1.80E-11 | FVC | 5.30E-05 | 22.366337 | Variant |
| rs6902771 | T | C | 0.463035 | 0.0139043 | 0.00164169 | 421986 | 2.50E-17 | FVC | 8.50E-05 | 35.871689 | Variant |
| rs6906411 | A | G | 0.449499 | -0.00953023 | 0.00165975 | 421986 | 9.40E-09 | FVC | 3.90E-05 | 16.458018 | Variant |
| rs6907284 | C | T | 0.697327 | -0.0117269 | 0.00179869 | 421986 | 7.00E-11 | FVC | 4.30E-05 | 18.146092 | Outlier |
| rs6919321 | A | G | 0.60753 | 0.0108866 | 0.00168563 | 421986 | 1.10E-10 | FVC | 4.70E-05 | 19.83418 | Variant |
| rs6923431 | C | A | 0.734557 | 0.0148601 | 0.00184047 | 421986 | 6.80E-16 | FVC | 6.00E-05 | 25.320559 | Variant |
| rs6937121 | G | T | 0.289184 | -0.0233431 | 0.00180879 | 421986 | 4.20E-38 | FVC | 0.000162 | 68.372484 | Variant |
| rs6965423 | C | T | 0.50901 | -0.0108395 | 0.00164539 | 421986 | 4.50E-11 | FVC | 5.10E-05 | 21.522282 | Variant |
| rs6977081 | T | G | 0.333825 | 0.0158604 | 0.00175433 | 421986 | 1.60E-19 | FVC | 8.60E-05 | 36.293745 | Variant |

| | | | | | | | | | | | |
|---|---|---|---|---|---|---|---|---|---|---|---|
| rs698748 | G | A | 0.581469 | 0.00936006 | 0.00167978 | 421986 | 2.50E-08 | FVC | 3.60E-05 | 15.191971 | Variant |
| rs7012637 | A | G | 0.4738 | -0.0101349 | 0.00165698 | 421986 | 9.60E-10 | FVC | 4.40E-05 | 18.568113 | Variant |
| rs7027103 | C | G | 0.203801 | -0.0165277 | 0.00204156 | 421986 | 5.70E-16 | FVC | 5.00E-05 | 21.100255 | Variant |
| rs7029491 | G | C | 0.334337 | 0.0134513 | 0.0017552 | 421986 | 1.80E-14 | FVC | 6.20E-05 | 26.16463 | Variant |
| rs7105777 | T | C | 0.631869 | -0.0108209 | 0.00170784 | 421986 | 2.40E-10 | FVC | 4.40E-05 | 18.568113 | Variant |
| rs7172622 | G | A | 0.566235 | 0.00937445 | 0.00169396 | 421986 | 3.10E-08 | FVC | 3.60E-05 | 15.191971 | Variant |
| rs723149 | G | A | 0.56266 | -0.014312 | 0.00165522 | 421986 | 5.30E-18 | FVC | 8.70E-05 | 36.715802 | Variant |
| rs723588 | C | T | 0.142475 | 0.0194088 | 0.00234291 | 421986 | 1.20E-16 | FVC | 4.00E-05 | 16.880035 | Outlier |
| rs72643433 | A | G | 0.252489 | -0.0198047 | 0.00189404 | 421986 | 1.40E-25 | FVC | 9.80E-05 | 41.358485 | Variant |
| rs72656010 | C | T | 0.132118 | -0.0309087 | 0.00243307 | 421986 | 5.60E-37 | FVC | 8.80E-05 | 37.13786 | Variant |
| rs72673891 | G | A | 0.067566 | 0.0325497 | 0.00328044 | 421986 | 3.30E-23 | FVC | 2.90E-05 | 12.237891 | Outlier |
| rs72755233 | A | G | 0.111547 | -0.0339278 | 0.00260619 | 421986 | 9.60E-39 | FVC | 8.00E-05 | 33.761421 | Variant |
| rs72976986 | A | G | 0.190197 | 0.0157335 | 0.00211371 | 421986 | 9.80E-14 | FVC | 4.00E-05 | 16.880035 | Variant |
| rs73125628 | T | C | 0.27862 | -0.0107868 | 0.00183637 | 421986 | 4.30E-09 | FVC | 3.30E-05 | 13.925932 | Variant |
| rs735830 | A | G | 0.208865 | -0.0162617 | 0.00202148 | 421986 | 8.70E-16 | FVC | 5.10E-05 | 21.522282 | Variant |
| rs7359156 | G | A | 0.209384 | -0.0137136 | 0.00202651 | 421986 | 1.30E-11 | FVC | 3.60E-05 | 15.191971 | Variant |
| rs74483355 | T | C | 0.112455 | -0.0195417 | 0.00260367 | 421986 | 6.10E-14 | FVC | 2.70E-05 | 11.393876 | Outlier |
| rs74767794 | G | A | 0.316352 | 0.0116672 | 0.00177224 | 421986 | 4.60E-11 | FVC | 4.40E-05 | 18.568113 | Variant |
| rs75166711 | A | G | 0.144555 | 0.0154071 | 0.00234799 | 421986 | 5.30E-11 | FVC | 2.50E-05 | 10.549864 | Variant |
| rs75421844 | C | T | 0.083714 | 0.0271346 | 0.00297229 | 421986 | 6.90E-20 | FVC | 3.00E-05 | 12.6599 | Variant |
| rs7562385 | T | C | 0.895666 | 0.0207069 | 0.00267966 | 421986 | 1.10E-14 | FVC | 2.60E-05 | 10.971869 | Variant |
| rs7632 | C | T | 0.512981 | -0.0104067 | 0.0016401 | 421986 | 2.20E-10 | FVC | 4.80E-05 | 20.256204 | Variant |
| rs7638143 | A | G | 0.582086 | -0.0139644 | 0.00166448 | 421986 | 4.90E-17 | FVC | 8.10E-05 | 34.183473 | Variant |
| rs76563195 | G | C | 0.242852 | -0.0142082 | 0.00192116 | 421986 | 1.40E-13 | FVC | 4.80E-05 | 20.256204 | Variant |
| rs77328429 | A | G | 0.114012 | 0.0188547 | 0.00257478 | 421986 | 2.40E-13 | FVC | 2.60E-05 | 10.971869 | Variant |
| rs77804065 | T | C | 0.224606 | -0.0293446 | 0.00198179 | 421986 | 1.30E-49 | FVC | 0.000181 | 76.392931 | Variant |
| rs7815955 | T | A | 0.203201 | -0.0239073 | 0.00204165 | 421986 | 1.10E-31 | FVC | 0.000105 | 44.312973 | Variant |
| rs784808 | C | T | 0.556276 | 0.0100268 | 0.00165571 | 421986 | 1.40E-09 | FVC | 4.30E-05 | 18.146092 | Variant |
| rs7854962 | G | C | 0.212959 | -0.017877 | 0.00202336 | 421986 | 1.00E-18 | FVC | 6.20E-05 | 26.16463 | Variant |
| rs78579285 | T | C | 0.181123 | -0.0147405 | 0.00213123 | 421986 | 4.60E-12 | FVC | 3.40E-05 | 14.347944 | Variant |
| rs7907533 | A | C | 0.548538 | 0.00988224 | 0.0016501 | 421986 | 2.10E-09 | FVC | 4.20E-05 | 17.724072 | Variant |
| rs7916879 | A | G | 0.369335 | -0.0108696 | 0.00170511 | 421986 | 1.80E-10 | FVC | 4.50E-05 | 18.990135 | Variant |
| rs7968682 | T | G | 0.517742 | -0.0246342 | 0.00164702 | 421986 | 1.40E-50 | FVC | 0.000265 | 111.8554 | Variant |
| rs7980065 | G | T | 0.43574 | -0.0106182 | 0.00165768 | 421986 | 1.50E-10 | FVC | 4.80E-05 | 20.256204 | Variant |
| rs8001781 | G | A | 0.661562 | -0.0118573 | 0.00173947 | 421986 | 9.30E-12 | FVC | 4.90E-05 | 20.678229 | Variant |
| rs8033776 | G | A | 0.4683 | 0.0109236 | 0.00164896 | 421986 | 3.50E-11 | FVC | 5.20E-05 | 21.944309 | Variant |
| rs803765 | A | C | 0.346274 | -0.013468 | 0.00172756 | 421986 | 6.40E-15 | FVC | 6.50E-05 | 27.430743 | Outlier |
| rs8064926 | G | A | 0.208126 | -0.0120312 | 0.00202461 | 421986 | 2.80E-09 | FVC | 2.80E-05 | 11.815883 | Variant |
| rs8107719 | C | T | 0.664833 | -0.0130467 | 0.00174248 | 421986 | 7.00E-14 | FVC | 5.90E-05 | 24.898525 | Variant |
| rs848606 | T | G | 0.32697 | -0.013659 | 0.00175059 | 421986 | 6.10E-15 | FVC | 6.30E-05 | 26.586667 | Variant |
| rs876531 | T | C | 0.455993 | -0.01839 | 0.00165098 | 421986 | 8.10E-29 | FVC | 0.000146 | 61.61866 | Variant |
| rs879098 | G | A | 0.39884 | -0.010048 | 0.00167973 | 421986 | 2.20E-09 | FVC | 4.10E-05 | 17.302053 | Variant |
| rs880610 | A | G | 0.166836 | -0.0152687 | 0.00220268 | 421986 | 4.20E-12 | FVC | 3.20E-05 | 13.50392 | Variant |
| rs9379084 | A | G | 0.115664 | -0.0251572 | 0.00264155 | 421986 | 1.70E-21 | FVC | 4.40E-05 | 18.568113 | Variant |
| rs9391253 | T | A | 0.32 | 0.0117856 | 0.00175829 | 421986 | 2.00E-11 | FVC | 4.60E-05 | 19.412157 | Variant |
| rs9435731 | A | C | 0.519025 | 0.0236663 | 0.0016398 | 421986 | 3.20E-47 | FVC | 0.000246 | 103.83361 | Variant |
| rs9496317 | C | T | 0.441344 | -0.0106374 | 0.00165069 | 421986 | 1.20E-10 | FVC | 4.90E-05 | 20.678229 | Variant |

| | | | | | | | | | | | |
|---|---|---|---|---|---|---|---|---|---|---|---|
| rs9745989 | C | T | 0.62791 | 0.0107594 | 0.00174129 | 421986 | 6.50E-10 | FVC | 4.20E-05 | 17.724072 | Variant |
| rs9821691 | T | G | 0.428996 | -0.0126874 | 0.00167142 | 421986 | 3.20E-14 | FVC | 6.70E-05 | 28.274822 | Outlier |
| rs985296 | A | G | 0.385446 | -0.0110356 | 0.00168371 | 421986 | 5.60E-11 | FVC | 4.80E-05 | 20.256204 | Variant |
| rs9886260 | A | G | 0.722783 | -0.0149214 | 0.00183802 | 421986 | 4.70E-16 | FVC | 6.30E-05 | 26.586667 | Variant |
| rs9988963 | G | C | 0.737251 | 0.0135266 | 0.00186634 | 421986 | 4.20E-13 | FVC | 4.80E-05 | 20.256204 | Variant |

FVC, forced vital capacity; SNP, single nucleotide polymorphism; IVW, inverse-variance weighted.

**Table S4.** Genetic instruments for COPD.

| SNP | Effect allele | Other allele | Effect allele frequency | Beta | Standard error | Sample size | P value | Exposure | R2 | F statistic | Outliers (Radial IVW) |
|---|---|---|---|---|---|---|---|---|---|---|---|
| rs10037493 | C | T | 0.55 | 0.122217633 | 0.010162937 | 257811 | 2.60E-33 | COPD | 0.000278 | 71.690832 | Variant |
| rs10114763 | T | A | 0.42 | 0.067658648 | 0.009463212 | 257811 | 8.70E-13 | COPD | 9.70E-05 | 25.009899 | Variant |
| rs10760580 | G | A | 0.71 | 0.067658648 | 0.01050709 | 257811 | 1.20E-10 | COPD | 6.60E-05 | 17.016517 | Variant |
| rs10866659 | G | A | 0.35 | 0.086177696 | 0.010404027 | 257811 | 1.20E-16 | COPD | 0.000121 | 31.198664 | Outlier |
| rs11049386 | T | A | 0.71 | 0.058268908 | 0.010480294 | 257811 | 2.70E-08 | COPD | 4.90E-05 | 12.63326 | Variant |
| rs11118406 | T | A | 0.28 | 0.076961041 | 0.011660025 | 257811 | 4.10E-11 | COPD | 6.80E-05 | 17.532204 | Outlier |
| rs11579382 | C | G | 0.42 | 0.058268908 | 0.010040551 | 257811 | 6.50E-09 | COPD | 6.40E-05 | 16.500832 | Variant |
| rs11655567 | C | T | 0.49 | 0.058268908 | 0.009701568 | 257811 | 1.90E-09 | COPD | 7.00E-05 | 18.047893 | Variant |
| rs117261012 | G | A | 0.16 | 0.086177696 | 0.013970915 | 257811 | 6.90E-10 | COPD | 4.00E-05 | 10.312773 | Variant |
| rs12373142 | G | C | 0.22 | 0.076961041 | 0.012593825 | 257811 | 9.90E-10 | COPD | 5.00E-05 | 12.891095 | Variant |
| rs12466981 | C | T | 0.73 | 0.058268908 | 0.010681936 | 257811 | 4.90E-08 | COPD | 4.50E-05 | 11.601927 | Variant |
| rs12519165 | A | T | 0.38 | 0.067658648 | 0.011102175 | 257811 | 1.10E-09 | COPD | 6.80E-05 | 17.532204 | Variant |
| rs13073544 | C | G | 0.28 | 0.058268908 | 0.010382911 | 257811 | 2.00E-08 | COPD | 4.90E-05 | 12.63326 | Variant |
| rs13140176 | A | G | 0.61 | 0.165514438 | 0.010208924 | 257811 | 4.10E-59 | COPD | 0.000485 | 125.098038 | Variant |
| rs1334576 | A | G | 0.42 | 0.058268908 | 0.010223107 | 257811 | 1.20E-08 | COPD | 6.10E-05 | 15.727308 | Variant |
| rs1441358 | G | T | 0.34 | 0.122217633 | 0.010236735 | 257811 | 7.40E-33 | COPD | 0.000248 | 63.952492 | Outlier |
| rs1529672 | C | A | 0.83 | 0.086177696 | 0.012913675 | 257811 | 2.50E-11 | COPD | 4.90E-05 | 12.63326 | Variant |
| rs153916 | T | C | 0.55 | 0.058268908 | 0.009424453 | 257811 | 6.30E-10 | COPD | 7.30E-05 | 18.821431 | Variant |
| rs1551943 | A | G | 0.23 | 0.076961041 | 0.012421542 | 257811 | 5.80E-10 | COPD | 5.30E-05 | 13.664601 | Variant |
| rs1570221 | A | G | 0.35 | 0.058268908 | 0.010413549 | 257811 | 2.20E-08 | COPD | 5.50E-05 | 14.180275 | Variant |
| rs16825267 | C | G | 0.92 | 0.173953307 | 0.018757945 | 257811 | 1.80E-20 | COPD | 4.90E-05 | 12.63326 | Variant |
| rs17759204 | G | A | 0.27 | 0.067658648 | 0.010431091 | 257811 | 8.80E-11 | COPD | 6.40E-05 | 16.500832 | Variant |
| rs2040732 | C | T | 0.58 | 0.058268908 | 0.010057912 | 257811 | 6.90E-09 | COPD | 6.30E-05 | 16.24299 | Variant |
| rs2096468 | A | C | 0.45 | 0.058268908 | 0.010611998 | 257811 | 4.00E-08 | COPD | 5.80E-05 | 14.953789 | Outlier |
| rs2284174 | C | T | 0.22 | 0.113328685 | 0.011929404 | 257811 | 2.10E-21 | COPD | 0.00012 | 30.940793 | Variant |
| rs2442776 | G | A | 0.15 | 0.086177696 | 0.013547096 | 257811 | 2.00E-10 | COPD | 4.00E-05 | 10.312773 | Variant |
| rs2571445 | A | G | 0.39 | 0.067658648 | 0.009720912 | 257811 | 3.40E-12 | COPD | 8.90E-05 | 22.947043 | Outlier |
| rs2579762 | C | A | 0.47 | 0.058268908 | 0.009218413 | 257811 | 2.60E-10 | COPD | 7.70E-05 | 19.852822 | Variant |
| rs2806356 | C | T | 0.18 | 0.09531018 | 0.012072013 | 257811 | 2.90E-15 | COPD | 7.10E-05 | 18.305739 | Variant |
| rs2897075 | C | T | 0.63 | 0.067658648 | 0.010984535 | 257811 | 7.30E-10 | COPD | 6.90E-05 | 17.790049 | Variant |
| rs34712979 | A | G | 0.25 | 0.165514438 | 0.011592253 | 257811 | 3.00E-46 | COPD | 0.000297 | 76.592021 | Variant |
| rs4585380 | G | A | 0.74 | 0.067658648 | 0.011446192 | 257811 | 3.40E-09 | COPD | 5.20E-05 | 13.406765 | Variant |
| rs4660861 | G | T | 0.57 | 0.058268908 | 0.010644676 | 257811 | 4.40E-08 | COPD | 5.70E-05 | 14.695951 | Outlier |
| rs4757118 | T | C | 0.54 | 0.058268908 | 0.009888368 | 257811 | 3.80E-09 | COPD | 6.70E-05 | 17.27436 | Outlier |
| rs55676755 | G | C | 0.34 | 0.104360015 | 0.00983673 | 257811 | 2.70E-26 | COPD | 0.000196 | 50.54047 | Variant |
| rs56134392 | C | T | 0.35 | 0.058268908 | 0.010652424 | 257811 | 4.50E-08 | COPD | 5.30E-05 | 13.664601 | Variant |
| rs62191105 | C | T | 0.8 | 0.086177696 | 0.012314958 | 257811 | 2.60E-12 | COPD | 6.10E-05 | 15.727308 | Variant |
| rs629619 | T | C | 0.2 | 0.076961041 | 0.012208224 | 257811 | 2.90E-10 | COPD | 4.90E-05 | 12.63326 | Outlier |
| rs646695 | C | T | 0.24 | 0.076961041 | 0.011690275 | 257811 | 4.60E-11 | COPD | 6.10E-05 | 15.727308 | Variant |
| rs647097 | C | T | 0.27 | 0.076961041 | 0.011306988 | 257811 | 1.00E-11 | COPD | 7.10E-05 | 18.305739 | Variant |
| rs674621 | C | T | 0.32 | 0.058268908 | 0.01008619 | 257811 | 7.60E-09 | COPD | 5.60E-05 | 14.438113 | Variant |
| rs7068966 | C | T | 0.49 | 0.09531018 | 0.009666268 | 257811 | 6.20E-23 | COPD | 0.000188 | 48.477206 | Variant |

| | | | | | | | | | | | |
|---|---|---|---|---|---|---|---|---|---|---|---|
| rs72626215 | G | A | 0.73 | 0.067658648 | 0.011231235 | 257811 | 1.70E-09 | COPD | 5.50E-05 | 14.180275 | Variant |
| rs72699855 | G | C | 0.81 | 0.076961041 | 0.013146831 | 257811 | 4.80E-09 | COPD | 4.10E-05 | 10.570602 | Outlier |
| rs7307510 | C | T | 0.81 | 0.076961041 | 0.01292367 | 257811 | 2.60E-09 | COPD | 4.20E-05 | 10.828433 | Variant |
| rs73158393 | C | G | 0.74 | 0.067658648 | 0.011715991 | 257811 | 7.70E-09 | COPD | 5.00E-05 | 12.891095 | Variant |
| rs7642001 | A | G | 0.37 | 0.076961041 | 0.009959851 | 257811 | 1.10E-14 | COPD | 0.000108 | 27.846379 | Variant |
| rs7650602 | C | T | 0.45 | 0.058268908 | 0.010681936 | 257811 | 4.90E-08 | COPD | 5.70E-05 | 14.695951 | Variant |
| rs7671261 | A | G | 0.55 | 0.086177696 | 0.009795794 | 257811 | 1.40E-18 | COPD | 0.000149 | 38.419265 | Variant |
| rs76841360 | A | G | 0.23 | 0.076961041 | 0.012374939 | 257811 | 5.00E-10 | COPD | 5.30E-05 | 13.664601 | Variant |
| rs7866939 | C | T | 0.33 | 0.058268908 | 0.010331274 | 257811 | 1.70E-08 | COPD | 5.50E-05 | 14.180275 | Variant |
| rs7958945 | G | A | 0.36 | 0.058268908 | 0.009537567 | 257811 | 1.00E-09 | COPD | 6.70E-05 | 17.27436 | Variant |
| rs798565 | G | A | 0.71 | 0.067658648 | 0.011490198 | 257811 | 3.90E-09 | COPD | 5.50E-05 | 14.180275 | Variant |
| rs803923 | A | G | 0.53 | 0.058268908 | 0.010480294 | 257811 | 2.70E-08 | COPD | 6.00E-05 | 15.469468 | Variant |
| rs9350191 | T | C | 0.85 | 0.113328685 | 0.015051623 | 257811 | 5.10E-14 | COPD | 5.60E-05 | 14.438113 | Variant |
| rs9399401 | T | C | 0.72 | 0.148420005 | 0.011136304 | 257811 | 1.60E-40 | COPD | 0.000278 | 71.690832 | Variant |
| rs9435731 | A | C | 0.51 | 0.058268908 | 0.009435663 | 257811 | 6.60E-10 | COPD | 7.40E-05 | 19.079278 | Variant |
| rs9525927 | G | A | 0.19 | 0.076961041 | 0.012950052 | 257811 | 2.80E-09 | COPD | 4.20E-05 | 10.828433 | Variant |
| rs955277 | T | C | 0.61 | 0.067658648 | 0.010622764 | 257811 | 1.90E-10 | COPD | 7.50E-05 | 19.337125 | Variant |
| rs9617650 | G | C | 0.79 | 0.076961041 | 0.012335212 | 257811 | 4.40E-10 | COPD | 5.00E-05 | 12.891095 | Outlier |
| rs979453 | G | A | 0.34 | 0.058268908 | 0.010270565 | 257811 | 1.40E-08 | COPD | 5.60E-05 | 14.438113 | Variant |

COPD, chronic obstructive pulmonary disease; SNP, single nucleotide polymorphism; IVW, inverse-variance weighted.

**Table S5.** Associations of lung function and COPD with the odds of hearing impairment stratified by age and sex.

| Exposure | Age | | | Sex | | |
|---|---|---|---|---|---|---|
| | < 60 (n = 59,977) | ≥ 60 (n = 49,691) | $P$-interaction | Male (n = 50,674) | Female (n = 58,994) | $P$-interaction |
| FEV1 | 0.72 (0.67, 0.76) | 0.76 (0.72, 0.79) | 0.300 | 0.87 (0.83, 0.91) | 0.83 (0.79, 0.86) | 0.477 |
| FVC | 0.70 (0.65, 0.75) | 0.75 (0.71, 0.79) | 0.064 | 0.87 (0.83, 0.92) | 0.83 (0.79, 0.86) | 0.408 |
| COPD* | 1.03 (0.91, 1.18) | 1.15 (1.05, 1.25) | 0.506 | 1.09 (0.99, 1.21) | 1.10 (0.99, 1.23) | 0.864 |

Data are presented as odds ratios (95% confidence intervals).

Models were adjusted for age (not included in age-stratified analyses), sex (not included in sex-stratified analyses), Townsend deprivation index, qualifications, employment, smoking, drink frequency, body mass index, diabetes, cardiovascular diseases, and music and occupational noise exposure.

*COPD was defined as the FEV1/FVC ratio below the lower limit of normal.

FEV1, forced expiratory volume in one second; FVC, forced vital capacity; COPD, chronic obstructive pulmonary disease.

**Table S6.** Association of COPD (FEV1/FVC < 0.7) with the odds of hearing impairment (n = 109,668).

| COPD | Unadjusted OR (95% CI) | *P* | Adjusted* OR (95% CI) | *P* |
|---|---|---|---|---|
| FEV1/FVC < 0.7† | 1.39 (1.32, 1.46) | <0.001 | 1.07 (1.02, 1.13) | 0.011 |

*Adjusted for age, sex, Townsend deprivation index, qualifications, employment, smoking, drink frequency, body mass index, diabetes, cardiovascular diseases, and music and occupational noise exposure.

†For COPD (FEV1/FVC < 0.7), cases were 15,560 (14.2%), controls were 94,108 (85.8%).

COPD, chronic obstructive pulmonary disease; OR, odds ratio; CI, confidence interval; FEV1, forced expiratory volume in one second; FVC, forced vital capacity.

**Table S7.** Association of COPD with the odds of hearing impairment after excluding participants with a history of asthma (n = 96,160).

| Exposure | Unadjusted OR (95% CI) | *P* | Adjusted* OR (95% CI) | *P* |
|---|---|---|---|---|
| COPD (FEV1/FVC < LLN)† | 1.27 (1.17, 1.38) | <0.001 | 1.16 (1.06, 1.26) | <0.001 |

*Adjusted for age, sex, Townsend deprivation index, qualifications, employment, smoking, drink frequency, body mass index, diabetes, cardiovascular diseases, and music and occupational noise exposure.

†For COPD (FEV1/FVC < LLN), cases were 5705 (5.9%), controls were 90,455 (94.1%).

OR, odds ratio; CI, confidence interval; COPD, chronic obstructive pulmonary disease; FEV1, forced expiratory volume in one second; FVC, forced vital capacity; LLN, lower limit of normal.

**Table S8.** Associations of lung function and COPD with the odds of self-reported hearing difficulty (n = 333,082).

| **Exposure** | **Adjusted*<br>OR (95% CI)** | ***P*** |
|---|---|---|
| FEV1, per IQR | 0.94 (0.93, 0.96) | <0.001 |
| FVC, per IQR | 0.94 (0.93, 0.95) | <0.001 |
| COPD (FEV1/FVC < LLN)† | 1.01 (0.98, 1.03) | 0.532 |
| COPD (FEV1/FVC < 0.7)‡ | 1.00 (0.98, 1.02) | 0.993 |

The FEV1 of each IQR is equivalent to 1.04 liters.

The FVC of each IQR is equivalent to 1.36 liters.

*Adjusted for age, sex, Townsend deprivation index, qualifications, employment, smoking, drink frequency, body mass index, diabetes, cardiovascular diseases.

†For COPD (FEV1/FVC < LLN), cases were 28,146 (8.5%), controls were 304,936 (91.5%).

‡For COPD (FEV1/FVC < 0.7), cases were 50,639 (15.2%), controls were 282,443 (84.8%).

OR, odds ratio; CI, confidence interval; FEV1, forced expiratory volume in one second; FVC, forced vital capacity; IQR, interquartile range; COPD, chronic obstructive pulmonary disease; LLN, lower limit of normal.

**Table S9.** Associations of lung function and COPD with the odds of self-reported hearing difficulty after excluding participants with history of noise exposure (n = 299,670).

| **Exposure** | **Adjusted*** **OR (95% CI)** | ***P*** |
|---|---|---|
| FEV1, per IQR | 0.94 (0.93, 0.96) | <0.001 |
| FVC, per IQR | 0.94 (0.93, 0.96) | <0.001 |
| COPD (FEV1/FVC < LLN)† | 1.01 (0.98, 1.04) | 0.498 |
| COPD (FEV1/FVC < 0.7)‡ | 1.01 (0.99, 1.03) | 0.448 |

The FEV1 of each IQR is equivalent to 1.03 liters.

The FVC of each IQR is equivalent to 1.34 liters.

*Adjusted for age, sex, Townsend deprivation index, qualifications, employment, smoking, drink frequency, body mass index, diabetes, cardiovascular diseases.

†For COPD (FEV1/FVC < LLN), cases were 25,208 (8.4%), controls were 274,462 (91.6%).

‡For COPD (FEV1/FVC < 0.7), cases were 45,301 (15.1%), controls were 254,369 (84.9%).

OR, odds ratio; CI, confidence interval; FEV1, forced expiratory volume in one second; FVC, forced vital capacity; IQR, interquartile range; COPD, chronic obstructive pulmonary disease; LLN, lower limit of normal.

**Table S10.** Two-sample Mendelian randomization analyses of the associations of lung function and COPD with sensorineural hearing loss after excluding radial IVW outliers.

| Exposure | No. of SNPs | Method | OR (95% CI) | *P* |
|---|---|---|---|---|
| FEV1 | 182 | IVW | 0.92 (0.84, 1.01) | 0.095 |
| | | MR-Egger | 0.92 (0.67, 1.26) | 0.607 |
| | | Weighted median | 1.01 (0.87, 1.17) | 0.903 |
| FVC | 212 | IVW | 0.91 (0.83, 0.99) | 0.031 |
| | | MR-Egger | 0.88 (0.68, 1.14) | 0.328 |
| | | Weighted median | 0.94 (0.82, 1.08) | 0.380 |
| COPD | 51 | IVW | 0.97 (0.94, 1.01) | 0.118 |
| | | MR-Egger | 1.04 (0.95, 1.15) | 0.412 |
| | | Weighted median | 0.97 (0.92, 1.02) | 0.236 |

For lung function, the results were reported as ORs per standard deviation increase in FEV1 and FVC. No., number; SNPs, single nucleotide polymorphisms; OR, odds ratio; CI, confidence interval; IVW, inverse variance weighted; FEV1, forced expiratory volume in one second; FVC, forced vital capacity; COPD, chronic obstructive pulmonary disease.

**Table S11.** Two-sample Mendelian randomization analyses of the associations of lung function and COPD with sensorineural hearing loss before excluding radial IVW outliers.

| Exposure | No. of SNPs | Method | OR (95% CI) | *P* |
|---|---|---|---|---|
| FEV1 | 201 | IVW | 0.93 (0.84, 1.04) | 0.217 |
| | | MR-Egger | 0.94 (0.65, 1.37) | 0.757 |
| | | Weighted median | 1.01 (0.87, 1.17) | 0.900 |
| FVC | 235 | IVW | 0.91 (0.83, 0.999) | 0.049 |
| | | MR-Egger | 0.81 (0.61, 1.07) | 0.143 |
| | | Weighted median | 0.93 (0.82, 1.06) | 0.299 |
| COPD | 61 | IVW | 0.98 (0.94, 1.02) | 0.311 |
| | | MR-Egger | 0.97 (0.85, 1.11) | 0.666 |
| | | Weighted median | 0.97 (0.92, 1.02) | 0.206 |

For lung function, the results were reported as ORs per standard deviation increase in FEV1 and FVC. No., number; SNPs, single nucleotide polymorphisms; OR, odds ratio; CI, confidence interval; IVW, inverse variance weighted; FEV1, forced expiratory volume in one second; FVC, forced vital capacity; COPD, chronic obstructive pulmonary disease.

**Table S12.** Heterogeneity statistics before and after excluding radial IVW outliers.

| Exposure | Before | | After | |
|---|---|---|---|---|
| | Cochran's Q | *P* | Cochran's Q | *P* |
| FEV1 | 289.224 | <0.001 | 162.575 | 0.833 |
| FVC | 302.291 | 0.002 | 170.348 | 0.982 |
| COPD | 118.680 | <0.001 | 48.979 | 0.514 |

IVW, inverse variance weighted; FEV1, forced expiratory volume in one second; FVC, forced vital capacity; COPD, chronic obstructive pulmonary disease.

**Table S13.** Results of the Egger-intercept test using the two-sample Mendelian randomization analysis after excluding radial IVW outliers.

| **Exposure** | **Egger-intercept** | **SE** | ***P*** |
|---|---|---|---|
| FEV1 | 0.000 | 0.002 | 0.993 |
| FVC | 0.000 | 0.002 | 0.804 |
| COPD | -0.006 | 0.004 | 0.156 |

IVW, inverse variance weighted; SE, standard error; FEV1, forced expiratory volume in one second; FVC, forced vital capacity; COPD, chronic obstructive pulmonary disease.

**Table S14.** Results of the MR-PRESSO test using the two-sample Mendelian randomization analysis after excluding radial IVW outliers.

| Exposure | PRESSO_beta (sd) | *P*-PRESSO | PRESSO_outlier_corrected_beta (sd) | *P*-PRESSO_outlier_corrected | MR-PRESSO global test | | MR-PRESSO distortion test | |
|---|---|---|---|---|---|---|---|---|
| | | | | | RSSobs | *P* | beta | *P* |
| FEV1 | -0.082 (0.046) | 0.079 | NA | NA | 164.307 | 0.841 | NA | NA |
| FVC | -0.096 (0.040) | 0.017 | NA | NA | 171.997 | 0.982 | NA | NA |
| COPD | -0.026 (0.017) | 0.120 | NA | NA | 50.773 | 0.516 | NA | NA |

MR-PRESSO, Mendelian randomization pleiotropy residual sum and outlier; IVW, inverse variance weighted; RSSobs, observed residual sum of squares; FEV1, forced expiratory volume in one second; FVC, forced vital capacity; COPD, chronic obstructive pulmonary disease.

**Table S15.** Mendelian randomization Steiger directionality test for FVC.

| Lung function | No. of SNPs | SNPs R2 of exposure | SNPs R2 of outcome | Correct causal direction | Steiger *P* value |
|---|---|---|---|---|---|
| FVC | 212 | 0.0342 | 0.0005 | TRUE | 0 |

FVC, forced vital capacity; No., number; SNPs, single nucleotide polymorphisms.
